# The oesophageal adenocarcinoma tumour immune microenvironment dictates outcomes with different modalities of neoadjuvant therapy – results from the AGITG DOCTOR trial and the cancer evolution biobank

**DOI:** 10.3389/fimmu.2023.1220129

**Published:** 2023-10-12

**Authors:** James M. Lonie, Sandra Brosda, Vanessa F. Bonazzi, Lauren G. Aoude, Kalpana Patel, Ian Brown, Sowmya Sharma, Guy Lampe, Venkateswar Addala, Lambros T. Koufariotis, Scott Wood, Nicola Waddell, Riccardo Dolcetti, Andrew P. Barbour

**Affiliations:** ^1^ Surgical Oncology Group, Frazer Institute, The University of Queensland, Brisbane, QLD, Australia; ^2^ Faculty of Medicine, The University of Queensland, Brisbane, QLD, Australia; ^3^ Envoi Specialist Pathologists, Brisbane, QLD, Australia; ^4^ Department of Pathology, Royal Brisbane and Women's Hospital, Brisbane, QLD, Australia; ^5^ Medlab Pathology, Sydney, NSW, Australia; ^6^ Medical Genomics, QIMR Berghofer Medical Research Institute, Brisbane, QLD, Australia; ^7^ Department of Anatomical Pathology, Central Laboratory Pathology Queensland, Brisbane, QLD, Australia; ^8^ Translational and Clinical Immunotherapy, Peter MacCallum Cancer Centre, Melbourne, VIC, Australia; ^9^ Sir Peter MacCallum Department of Oncology, The University of Melbourne, Melbourne, VIC, Australia; ^10^ Department of Microbiology and Immunology, The University of Melbourne, Melbourne, VIC, Australia; ^11^ Department of Surgery, Princess Alexandra Hospital, Brisbane, QLD, Australia

**Keywords:** oesophageal adenocarcinoma, tumour microenvironment, neoadjuvant therapy, survival, treatment response

## Abstract

A plateau in treatment effect can be seen for the current ‘one-size-fits-all’ approach to oesophageal adenocarcinoma (OAC) management using neoadjuvant chemoradiotherapy (nCRT) or chemotherapy (nCT). In OAC, the tumour microenvironment (TME) is largely immunosuppressed, however a subgroup of patients with an immune-inflamed TME exist and show improved outcomes. We aimed to understand the overall immune-based mechanisms underlying treatment responses and patient outcomes in OAC, and in relation to neoadjuvant therapy modality. This study included 107 patients; 68 patients were enrolled in the Australian Gastro-Intestinal Trials Group sponsored DOCTOR Trial, and 38 patients were included from the Cancer Evolution Biobank. Matched pre-treatment and post-treatment tumour biopsies were used to perform multi-modality analysis of the OAC TME including NanoString mRNA expression analysis, multiplex and single colour immunohistochemistry (IHC), and peripheral blood mononuclear cell analysis of tumour-antigen specific T cell responses. Patients with the best clinicopathological outcomes and survival had an immune-inflamed TME enriched with anti-tumour immune cells and pathways. Those with the worst survival showed a myeloid T regulatory cell enriched TME, with decreased CD8^+^ cell infiltration and increased pro-tumour immune cells. Multiplex IHC analysis identified that high intra-tumoural infiltration of CD8^+^ cells, and low infiltration with CD163^+^ cells was associated with improved survival. High tumour core CD8^+^ T cell infiltration, and a low tumour margin infiltration of CD163^+^ cells was also associated with improved survival. nCRT showed improved survival compared with nCT for patients with low CD8^+^, or high CD163^+^ cell infiltration. Poly-functional T cell responses were seen with tumour-antigen specific T cells. Overall, our study supports the development of personalised therapeutic approaches based on the immune microenvironment in OAC. Patients with an immune-inflamed TME show favourable outcomes regardless of treatment modality. However, in those with an immunosuppressed TME with CD163^+^ cell infiltration, treatment with nCRT can improve outcomes. Our findings support previous studies into the TME of OAC and with more research, immune based biomarker selection of treatment modality may lead in improved outcomes in this deadly disease.

## 1 Introduction

Oesophageal adenocarcinoma (OAC) is a deadly disease with a rising incidence globally (1). For curative intent, standard of care involves neoadjuvant therapy (NAT) with chemotherapy (nCT), or chemoradiotherapy (nCRT) followed by surgery (2). NAT has increased survival over surgery alone (3–5), and despite significantly improved pathological response rates for nCRT over nCT, no overall survival (OS) difference has been demonstrated (6–8), with treatment choice largely influenced by regional preferences. Immune-checkpoint blockade (ICB) has revolutionised treatment for multiple cancer types (9), however the role of ICB in OAC is only beginning to emerge. Adjuvant ICB following nCRT and surgery has shown promising results (10), while Phase III trial results for adjuvant ICB following CT, and in the neoadjuvant setting are yet to be reported.

The presence of a pre-existing anti-tumour immune response has been shown to be a crucial determinant of the improved survival of patients treated with immunotherapy across multiple cancer types (11). Three broad immune phenotypes have been described that correlate with patient response to immunotherapy (12): immune-inflamed, characterised by intra-tumoural inflammation with evidence of an anti-tumour immune response; immune-excluded, characterised by immune cell abundance within the surrounding stroma, but not within the tumour parenchyma, and; immune-desert, characterised by absence or paucity of both tumoural and stromal immune infiltration. The complex interplay between tumour and immune cells within the tumour microenvironment (TME) of these phenotypes have been described previously as the immune contexture (13), with their prognostic relevance elegantly described in colorectal cancer by the Immunoscore (14). Our group and others have found OAC to have a largely immunosuppressive and immune-excluded TME (15, 16), with pro-tumour immune cells such as T regulatory cells (Tregs), and M2-macrophages shown to negatively affect treatment response and outcomes (17–20). A subgroup of patients do exist however that display an immune-inflamed TME, with good outcomes (21, 22).

Increasing evidence has shown that CT and radiotherapy (RT) can modulate the immune contexture of the TME (15, 23–25). Radiation and some chemotherapeutic agents have been reported to induce or enhance immunogenic cell death through various mechanisms such as release of immune stimulating molecules such as damage-associated molecular patterns and upregulation of tumour antigens and antigen presentation machinery (15). Most studies investigating the immune landscape of OAC have been performed in direct-to-surgery patients or nCRT treated patients (16–19, 26, 27), with only limited data available for nCT treated patients (28–30). Crucially, to date, no study has directly compared nCT and nCRT.

Understanding the immune-based mechanisms underlying clinicopathological outcomes and treatment-specific responses is foundation knowledge that is required to understand which immunotherapeutic strategies may be beneficial, and how best to tailor and sequence therapy. Here, we have exploited multiple complementary analyses to profile the TME of OAC and outline the effects on outcomes and the differences in the post-NAT TME based on treatment modality.

## 2 Materials and methods

### 2.1 Cohort and samples

Patients were recruited through the cancer evolution biobank (HREC/10/QPAH/152, UQ2011001287), and the AGITG DOCTOR trial (31) between 2009 and 2021. Study approval has been granted by the Metro South Health Research Ethics Committee (HREC/19/QMS/5554), The University of Queensland Ethics Committee (UQ2019002466), and the QIMR Berghofer Human Research Ethics Committee (HREC P3559). Written, informed consent was obtained from all patients. All patients were histologically proven to have oesophageal or gastroesophageal adenocarcinoma, were of curative intent, and underwent nCT or nCRT. Pathological response was assessed using major pathological response rate (MPR) (32). MPR was defined as tumour regression >90% using the Becker regression grading system (33) combined with N0 nodal stage. Non-MPR was defined as tumour regression <90% and/or N+ disease, or those with disease progression on NAT. Clinical data was recorded at the time of enrolment and follow-up data were used to determine clinicopathological outcomes (HREC/15/QPAH/614).

Pre-treatment biopsies (“pre”) were taken at endoscopy and post-treatment biopsies (“post”) were taken at surgery. Samples were placed in RNAlater™ prior to storage at -80°C or formalin-fixed and paraffin-embedded (FFPE). Haematoxylin and eosin (H&E) slides were independently assessed for tumour content and annotated for tumour, stroma, and normal regions by three experienced gastrointestinal pathologists who were blinded to clinical outcomes (IB, GL, SS). DNA and RNA were extracted using the Qiagen AllPrep DNA/RNA mini kit according to the manufacturers protocol (Qiagen, Germany). Pre-treatment Peripheral blood mononuclear cells (PBMCs) were isolated from whole-blood using standard Ficoll-Hypaque density centrifugation, and frozen at -80°C in 10% Foetal Bovine Serum (FBS) + 10% DMSO for 24-hours prior to long-term storage at -180°C.

### 2.2 NanoString mRNA expression analysis

RNA from 63 tumour biopsies (45 pre, and 18 matched post) was hybridised with the 770 gene nCounter^®^ PanCancer Immune Profiling Panel (Nanostring Technologies, USA). Data were analysed using NanoString nSolver software (version 4.0). One sample failed quality control (binding density QC) and was excluded from analysis. Data were normalised and analysed using nCounter^®^ Advanced Analysis Software (version 2.0). NanoString-curated gene-sets representing specific cell types and immune pathways were used. For differential gene expression analysis, genes with an absolute Log_2_ fold change >1.5 and an adjusted p < 0.05 were considered significant.

### 2.3 Multiplex immunofluorescence and data acquisition

Multiplex immunofluorescence (mIF) was performed on matched pre- and post-NAT FFPE sections from 95 patients. Staining was performed using the Opal™ 6-plex detection kit (Akoya Biosciences, USA). After deparaffinization, hydration, and antigen retrieval (CD8, FoxP3: Dako pH 9.0, 100°C, 20 minutes; CD163: Diva MW 15 minutes), sections were treated with peroxidase and blocked. Slides were stained using the Ventana Benchmark Ultra Slide Stainer (Roche Tissue Diagnostics, Switzerland). Antibodies used were: CD8 clone C8/144B (Dako #M7013, 1:1600, Mouse, Opal690); FoxP3 clone 22510 (Abcam #ab22510, 1:5000, Mouse, Opal570); CD163 clone 10D6 (Biocare Medical #CM353AK, 1:200, Mouse, Opal520). Nuclei were stained using DAPI.

Imaging was performed using the Vectra^®^ 3 automated quantitative pathology imaging system (Akoya Biosciences, USA). Whole slide scans were viewed using Phenochart^®^ (Akoya Biosciences, USA), and multispectral regions were selected and scanned at 20x magnification. Multispectral image analysis was performed using inForm^®^ software (Akoya Biosciences, USA). Tissue segmentation was performed for tumour epithelium, stroma, and normal epithelium using matched annotated H&E slides. Cell segmentation and cellular phenotyping were performed for each marker. Cell densities were calculated as cells per mm^2^.

To calculate tumour core (TC) and tumour margin (TM) immune cell infiltration, the location of each cell type was assessed using their distance from the infiltrative border of the tumour toward the centre of the tumour at 50 µm intervals. TC cells were defined as cells greater than the given distance from the tumour border towards the tumour centre (>50 µm, >100 µm and >150 µm), and TM cells were defined as cells within the given distance from the tumour border only (<50 µm, <100 µm, <150 µm). The proportion of the total number of cell type of interest to total cell number at each given distance was calculated.

### 2.4 PD-L1 immunohistochemistry staining and data acquisition

Immunohistochemistry was performed on matched pre- and post-NAT FFPE sections from 95 patients. After deparaffinization, hydration and antigen retrieval (Dako pH 9.0, 120°C, 8 minutes), sections were treated with peroxidase and then blocked. Slides were stained using PD-L1 clone E1L3N^®^ (Cell Signalling Technology #13684, 1:100, Rabbit), and bound antibody was detected using DAB. Human tonsil sections were stained as a positive control.

Whole slides were scanned at 20x magnification using an Olympus VS200 slide scanner (Olympus, Japan). The Visiopharm^®^ Image Analytical System (Version 2017.2.4.3387) was used to quantify staining (Visiopharm, Denmark). Regions of tumour and stroma were selected using matched annotated H&E slides and PD-L1 quantification was performed using the embedded Visiopharm workflow. PD-L1 staining levels were reported as percentage of marker positivity of the assessed area.

### 2.5 HLA-typing

HLA-typing was performed using available whole genome (WGS) or whole exome sequencing (WES) data as described in a previous study (22). Briefly, Class I HLA genotypes were determined for matched tumour-normal pairs using OptiType (v1.3.1) with default parameters (34).

### 2.6 Assessment of tumour-antigen-specific T cell responses

PBMCs from HLA-A*02 patients or healthy donors were thawed at 37°C and rested overnight in complete-RPMI (cRPMI: RPMI-1640 + 10% FBS + 1% penicillin/streptomycin; Gibco, USA) in a humidified incubator at 37°C, 5% CO_2_. 1-2 x 10^6^ PBMCs were cultured in cRPMI with tumour antigen epitopes presented by the common HLA-A*02:01 molecule: survivin (Sur1 M2, LMLGEFLKL), telomerase (hTERT-540, ILAKFLHWL), MAGE-A3 (MAGE-A3_271-279_, FLWGPRALV), NY-ESO-1 (NY-ESO-1_157-165_, SLLMWITQC) (all 10 µg/mL), and CytoStim™ (Miltenyi Biotec, Germany) or a CEF pool (2 µg/mL/peptide) and a mock condition (CRPMI + 0.5% DMSO) as positive and negative controls. PBMCs were cultured for 8 hours with 1µg/mL GolgiPlug™ (BD Biosciences, USA) and CD107a (AF488, 1:400, BD biosciences, USA), then kept at 4°C overnight prior to antibody staining and flow cytometry assessment. Cells were stained with LIVE/DEAD Aqua™ (1:1000, ThermoFisher, USA) for 20 minutes at room temperature (RT). Cell membrane staining was performed using CD3 (BUV395, 1:200, BD Biosciences), CD4 (BUV737, 1:150, BD Biosciences), and CD8 (BV785, 1:200, BD Biosciences) for 30 minutes at RT. Cells were fixed and permeabilised using CytoFix/CytoPerm^®^ (BD Biosciences) according to manufacturer’s instructions. Intracellular staining was then performed for IFNγ (BV421, 1:100, BD Biosciences), TNFα (PE, 1:50, BD Biosciences), and IL-2 (BV650, 1:40, BD Biosciences) for 30 minutes at 4°C. Data acquisition was performed on a LSRFortessa X20 cytometer (BD Biosciences). Downstream analyses were performed using FlowJo™ software (version ≥ 10.2, Tree Star Inc., USA). Due to limitations in available PBMC numbers, only one biological replicate was able to be performed.

### 2.7 Statistical analysis

All statistical analyses were performed using R statistical software (version ≥3.6.4), and RStudio (version ≥1.2.5033). Associations between categorical variables were performed using chi-square tests. Associations between continuous variables were performed using the Wilcoxon signed-rank test, or Kruskal-Wallis test. For survival analyses, variables were first dichotomised using maximally selected rank statistics (35) or defined cut-offs as detailed in figure legends. Dichotomised variables were compared using Kaplan-Meier estimates and log-rank tests. Hazard ratios (HR) were calculated using Cox proportional hazard models. A p-value <0.05 was considered statistically significant. The Benjamini-Hochberg procedure was utilised to correct for multiple testing. P values are reported as: p ≥ 0.05 (n.s., non-significant); *, p <0.05; **, p <0.01; ***, p <0.001; ****, p <0.0001.

## 3 Results

### 3.1 Patient cohort

107 patients were included in the study (demographic characteristics of these patients are described in **Supplementary Table S1**). 54/107 (50.5%) received nCRT, and 53/107 (49.55%) received nCT. Overall, 31/107 (29%) achieved MPR, and 59/107 (55%) of patients were pathologically lymph node negative (N0). Median progression free survival (PFS) was 21 months (range 2 – 89), and median OS was 25 months (range 4 – 89). Median follow up for survivors was 39 months (range 4 – 89).

### 3.2 The pre-treatment immune landscape in OAC

**Figure 1:**
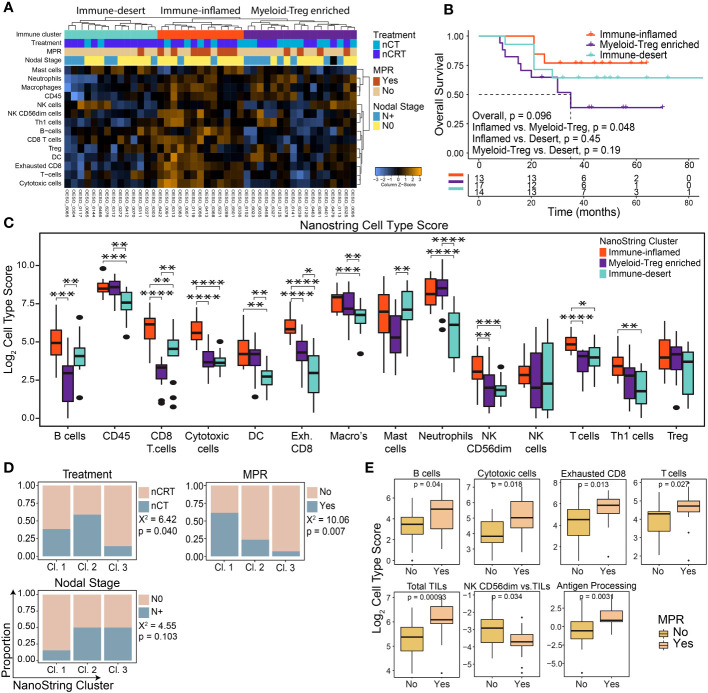
The pre-treatment immune landscape of OAC using mRNA expression. NanoString mRNA expression profiling was used to profile the TME in 44 pre-treatment OAC samples. **(A)** Unsupervised hierarchical clustering of NanoString-derived cell types revealed three clusters: Immune-inflamed, Myeloid-Treg enriched, and Immune-desert. The top bars show the immune clusters and clinicopathological variables. **(B)** Overall survival for each cluster showed the best survival for Immune-inflamed, and worst survival for Myeloid-Treg enriched. **(C)** Significant differences were seen between NanoString cell type scores between each immune cluster. **(D)** Significant differences were seen between clinicopathological variables for each immune cluster. **(E)** MPR was associated with NanoString-derived cell type scores and pathways. Survival analyses were performed using Kaplan-Meier survival estimates with log-rank tests. Boxplots show the median (centre bar), the 1^st^ and 3^rd^ quartiles (upper and lower limit of boxes), and the largest and smallest value that is ≤ 1.5 times the interquartile range (whiskers). Comparisons were made using Wilcoxon rank-sum tests, or Chi-square tests as appropriate. P values are indicated or represented by: n.s., non-significant; *p <0.05; **p <0.01; ***p <0.001; ****p <0.0001. Cl, cluster; nCRT, neoadjuvant chemoradiotherapy; nCT, neoadjuvant chemotherapy; DC, dendritic cell; MPR, major pathological response; NK, natural killer; Treg, T regulatory cell.

NanoString mRNA gene expression was used to profile the TME of 44 patients: 17/44 (38.6%) were treated with nCT and 27/44 (61.4%) with nCRT. Unsupervised hierarchical clustering of cell-type scores revealed three immune clusters: immune-inflamed, myeloid-Treg enriched, and immune-desert (**Figure 1A**). Differences in OS between clusters trended towards significance, with patients with immune-inflamed tumours showing significantly improved survival compared to those with myeloid-Treg enriched tumours (p = 0.048; **Figure 1B**).

Immune-inflamed tumours showed higher enrichment of immune cells with anti-tumour potential (including T cells, CD8^+^ T cells, B cells, natural killer cells, and Th1 cells) compared to the other two clusters (**Figure 1C**) and had significantly higher scores related to relative anti-tumour cell scores and signalling pathways (**Supplementary Figures S1A, B**).”. Myeloid-Treg enriched OACs had high infiltration of CD45^+^ cells mainly with immunosuppressive properties, including macrophages, neutrophils, and Tregs, with the lowest CD8^+^ T cell and B cell infiltration and lowest ratio of anti-tumour immune cells (**Figure 1C**; **Supplementary Figure S1A**). Immune-desert OACs had generally lower immune cell infiltration with the lowest levels of exhausted CD8^+^ T cells, macrophages, and neutrophils of the three clusters. However, these tumours displayed the highest ratio of B cells to tumour infiltrating lymphocytes (TILs), and CD8^+^ T cells to exhausted T cells (**Supplementary Figure S1A**).

Congruent with OS and immune infiltration findings, the immune-inflamed group had the highest proportion of N0 (p = 0.103) and MPR (p = 0.007) patients following NAT (**Figure 1D**). Interestingly, although the immune-desert cluster had a significantly higher proportion of patients treated with nCRT, they were least likely to achieve MPR (**Figure 1D**). Patients with MPR showed a profile consistent with higher anti-tumour cellular responses, along with higher antigen processing and cytotoxicity pathway scores, while non-MPR patients showed higher numbers of mature, cytolytic, weakly cytokine producing CD56^dim^ NK cells compared to total TILs (p = 0.034; **Figure 1E**). Differential gene expression analysis comparing MPR and non-MPR cases revealed 9 differentially expressed genes (**Supplementary Figure S1C**): the 8 upregulated genes are involved in adaptive immune cell recruitment and activation including *TNFSF13B*, *Ly9*, *CXCL11*/*CXCR3* and *SLAMF7*. Interestingly, *CCL18* was also upregulated in MPRs. CCL18 is secreted by tumour-associated macrophages and was associated with cancer progression in other cancer types (36, 37). The Treg chemoattractant CCL22 was downregulated in MPRs. Overall, these findings indicate an increased likelihood of treatment response in patients with a functional, immune-inflamed TME.

### 3.3 Spatial landscape of immune infiltration in OAC

**Figure 2:**
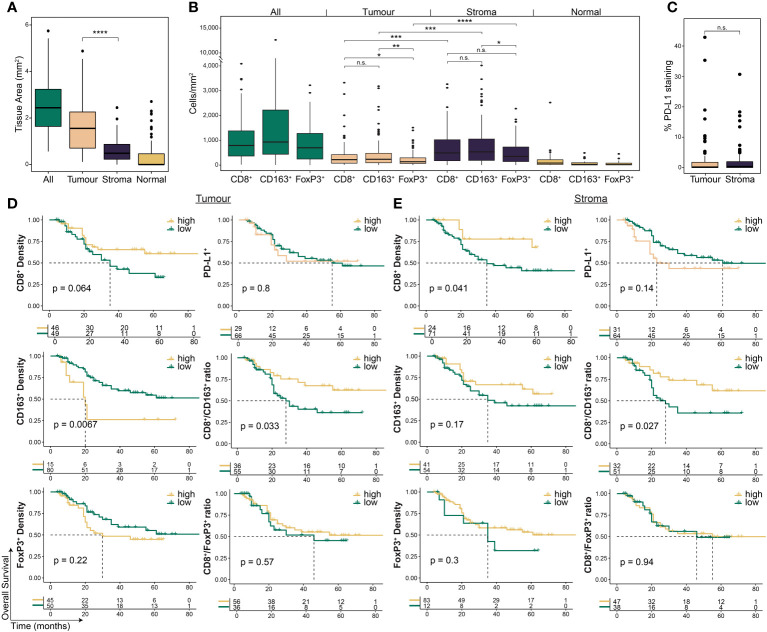
The pre-treatment spatial immune landscape of OAC and associated survival outcomes using multiplex/single colour immunohistochemistry. Multiplex/single stain IHC of 95 pre-NAT samples was used to profile the spatial distribution of CD8^+^ cells, CD163^+^ cells, FoxP3^+^ cells and PD-L1^+^ cells. **(A)** The area of tumour tissue was significantly higher than stroma. **(B)** A significantly higher density of immune cells was seen in the stromal compartment compared to tumour. In tumour a higher density of CD8 ^+^ cells and CD163 ^+^ cells was seen compared to FoxP3 ^+^ cells, while no difference was seen between CD8 ^+^ and CD163 ^+^ cells. In stroma only a higher density of CD163^+^ cells was seen compared to FoxP3 ^+^ cells. **(C)** No difference was seen in PD-L1 expression between tumour and stroma. PD-L1 staining is reported at the percentage area of marker activity for a total region. **(D)** Overall survival based on intra-tumoural cell density, PD-L1^+^ expression, or cell type ratios. **(E)** Overall survival based on stromal cell density, PD-L1^+^ expression, or cell type ratios. For survival estimate cut-offs, maximally selected rank statistics were used to dichotomise patients into high and low groups. For PD-L1 staining a cut-off of 1% was used. For cell type ratios a cut-off of 1 was used to dichotomise patients into high and low groups. Survival analyses were performed using Kaplan-Meier survival estimates with log-rank tests. Boxplots show the median (centre bar), the 1^st^ and 3^rd^ quartiles (upper and lower limit of boxes), and the largest and smallest value that is ≤ 1.5 times the interquartile range (whiskers). Comparisons were made using Wilcoxon rank-sum tests. P values are indicated or represented by: n.s., non-significant; *p <0.05; **p <0.01; ***p <0.001; ****p <0.0001. IHC, immunohistochemistry; NAT, neoadjuvant therapy; n.s., non-significant.

To investigate the spatial contexture of the TME, we used a mIF panel in 95 tumours to focus on CD8^+^ T cells, FoxP3^+^ Treg cells, and CD163^+^ M2-macrophages, and single-stain IHC of PD-L1, which have been shown to influence therapy response and outcomes in OAC patient subgroups (17–19). Pre-treatment samples had an average tissue area of 2.67 mm^2^ (range 0.59 – 6.03; **Figure 2A**). The average area of tumour epithelium (“tumour”) in each sample was 1.67 mm^2^ (range 0.12 – 5.12), with an average area of peri-tumoural stroma (“stroma”) of 0.63 mm^2^ (range 0 – 2.57) and normal epithelium (“normal”) of 0.37 mm^2^ (range 0 – 2.84). There was a significantly greater area of tumour tissue compared to stroma (p < 0.001; **Figure 2A**). Median cell densities are shown in **Figure 2B**. In line with previous studies, the stromal compartment contained a higher density of all immune cell types compared to tumour (**Figure 2B**), providing further evidence for an overall immune-excluded phenotype of OAC (16, 17). For PD-L1 expression no difference was seen between compartments (**Figure 2C**).

### 3.4 Survival outcomes based on spatial immune cell distribution in pre-treatment samples

We assessed the correlation of immune cell markers with the survival of all patients investigated (**Figure 2D**, **Supplementary Table S2**). Intra-tumoural CD8^+^ cell density was associated with a trend towards improved OS (p = 0.064; **Figure 2D**), and higher stromal CD8^+^ cell density was associated with improved OS (p = 0.041; **Figure 2E**). CD163^+^ cell density in the tumour compartment (p = 0.0067), but not in stroma (p = 0.17), was associated with worse OS (**Figures 2D, E**). PFS was similar to OS for CD8^+^ and CD163^+^ cells (**Supplementary Table S3**). Neither FoxP3^+^ density or PD-L1 expression were associated with survival (**Figures 2D, E**; **Supplementary Table S3**).

To explore the relationship between CD8^+^ cells and immunosuppressive CD163^+^ and FoxP3^+^ cells, we next assessed their ratios within each compartment (17). High intra-tumoural CD8^+^/CD163^+^ ratio was associated with improved OS (HR 0.45, log-rank p = 0.029; **Figure 2D**) and PFS (HR 0.49, log-rank p = 0.044; **Supplementary Table 3**), and a high stromal ratio significantly correlated with better OS (HR 0.42, log-rank p = 0.03; **Figure 2E**) but not PFS (HR 0.57, log-rank p = 0.12; **Supplementary Table 3**). No significant association with survival was seen for the CD8^+^/FoxP3^+^ ratio (**Figures 2D, E**; **Supplementary Table 3**).

**Figure 3:**
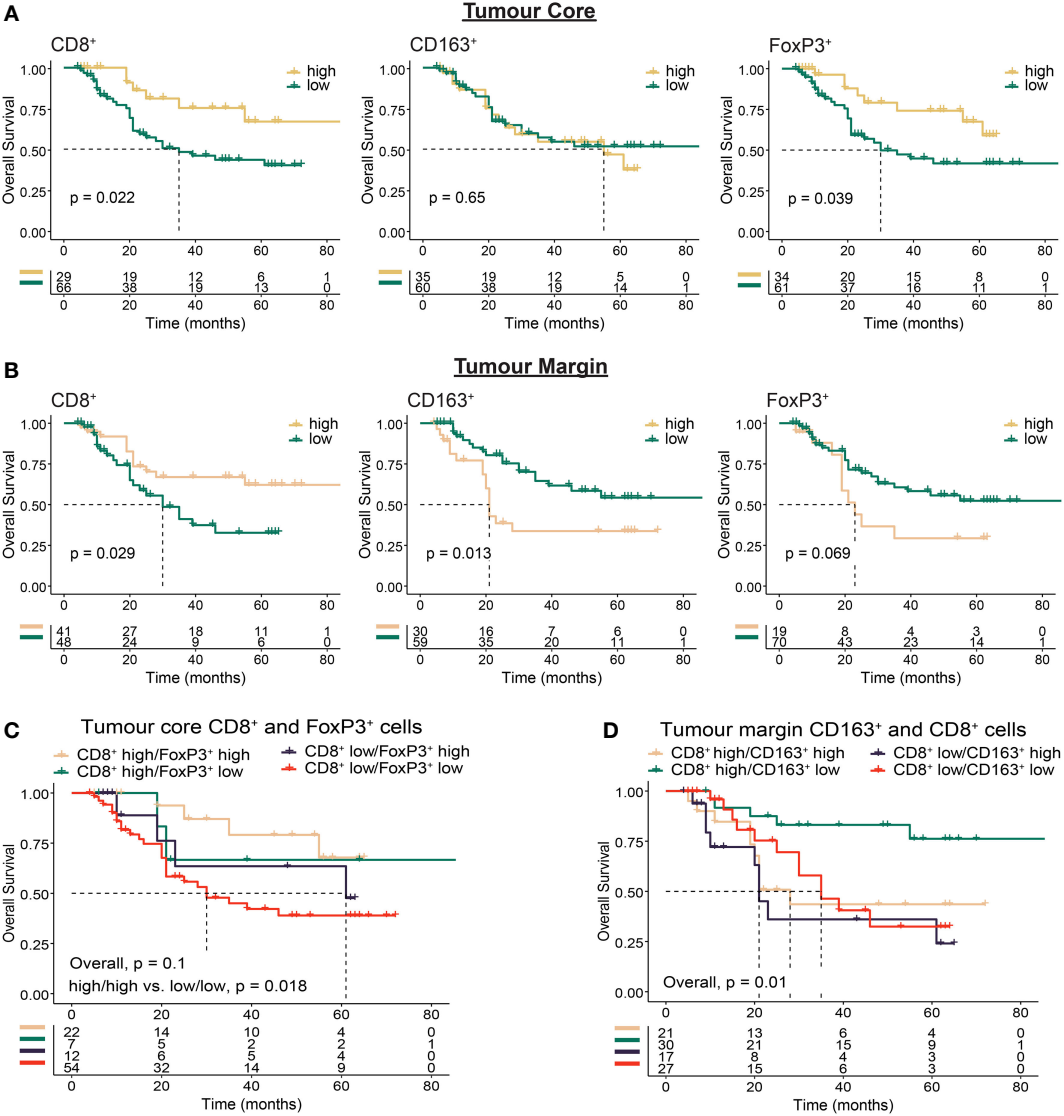
Immunohistochemistry based analysis of immune cell spatial distribution in the tumour core or at the tumour margin. Multiplex immunohistochemistry was used to determine the distance of immune cells from the tumour margin in OAC tumours. Cell location for each marker was assessed using 50 µm distances from tumour margin to determine the proportion of cells at a given distance from the tumour margin to the tumour core (tumour core cells), or within a given distance from the tumour margin (tumour margin cells). **(A)** Overall survival based on the proportion of tumour core immune cells >150 µm from the tumour margin. **(B)** Overall survival based on the proportion of tumour margin immune cells <50 µm from the tumour margin. **(C)** Overall survival based on co-infiltration of tumour core CD8^+^ and FoxP3^+^ cells. Dichotomised cell types were combined into high/high, high/low, low/high and low/low groups. **(D)** Overall survival based on co-infiltration of tumour margin CD8^+^ and CD163^+^ cells. Dichotomised cell types were combined into high/high, high/low, low/high and low/low groups. For survival estimate cut-offs, maximally selected rank statistics were used to dichotomise patients into high and low groups. Survival analyses were performed using Kaplan-Meier survival estimates with log-rank tests.

Next, we sought to determine whether the intra-tumour distribution of immune cells based on infiltration into the TC or location at the TM were associated with clinical outcomes (**Figure 3**, **Supplementary Figure S2**). We reasoned that infiltration of effector cells into the tumour core should favour improved outcomes, while immune excluded tumours should have a less favourable prognosis. We found high TC CD8^+^ (p = 0.022) and FoxP3^+^ (p = 0.039) cell infiltration was associated with improved survival (**Figure 3A**, **Supplementary Figure S2**). Further analysis revealed that patients with both high CD8^+^ and high FoxP3^+^ cell infiltration had the best outcomes, while low infiltration of both was the least favourable (p = 0.018; **Figure 3C**).

A high proportion of CD8^+^ cells at the TM was associated with OS (p = 0.029), while in contrast to the tumour core a high proportion of FoxP3+ cells at the TM was associated with impaired survival (p = 0.069; **Figure 3B**). Although intra-tumoural density of CD163^+^ cells was associated with impaired survival (**Figure 2D**), no survival difference was seen based on TC infiltration (p = 0.65, **Figure 3A**). When focussing on cells located at the TM however, we found high CD163^+^ correlated with worse OS (p = 0.013; **Figure 3B**). Combined with TM CD8^+^ cells, high TM CD8^+^/low CD163^+^ infiltration correlated with improved OS (p = 0.01; **Figure 3D**). These findings provide further evidence for the role of immunosuppressive cell types such as CD163^+^ and FoxP3^+^ cells in mediating local immunosuppression and provide evidence towards a potential mechanism of tumour exclusion facilitated by TM CD163^+^ cells.

### 3.5 Survival and pathological outcomes after stratification for treatment modality

**Figure 4:**
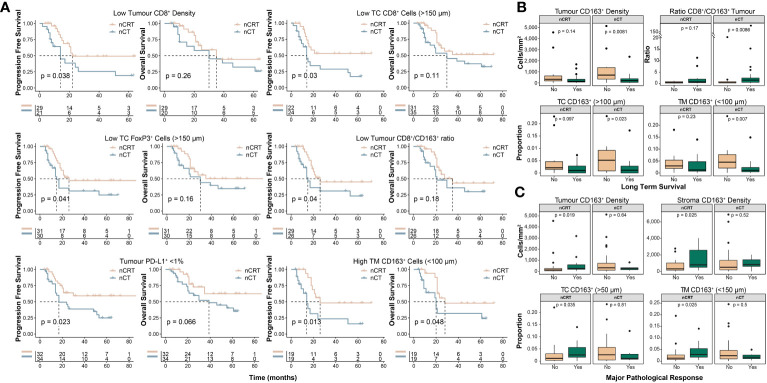
Clinicopathological outcomes based on the pre-treatment spatial immune landscape and neoadjuvant therapy type. Using the spatial immune landscape of pre-treatment samples, survival and clinicopathological outcomes were determined based on NAT-type. **(A)** Overall survival and progression free survival for low tumour CD8^+^ cell density, low tumour core CD8^+^ cells, low tumour core FoxP3^+^ cells, low tumour CD8^+^/CD163^+^ cell ratio, tumour PD-L1^+^ expression <1%, and high tumour margin CD163^+^ cells. **(B)** In patients treated with nCT, high intra-tumoural CD163^+^ cells results in significantly lower rates of long term survival, which was defined as survival ≥24 months. **(C)** In patients treated with nCRT, high tumoural and stromal CD163^+^ cells resulted in increased rates of MPR. For survival estimate cut-offs, the maximally selected rank statistic was used to dichotomise patients into high and low groups. For PD-L1^+^ staining a cut-off of 1% was used. Survival analyses were performed using Kaplan-Meier survival estimates with log-rank tests. Boxplots show the median (centre bar), the 1^st^ and 3^rd^ quartiles (upper and lower limit of boxes), and the largest and smallest value that is ≤ 1.5 times the interquartile range (whiskers). Comparisons were made using Wilcoxon rank-sum tests. IHC, immunohistochemistry; NAT, neoadjuvant therapy; nCRT, neoadjuvant chemoradiotherapy; nCT, neoadjuvant chemotherapy; TC, tumour core; TM, tumour margin.

We next undertook exploratory analyses examining how NAT-modality affects survival and MPR based on the pre-treatment immune landscape. Although treatment-specific survival outcomes generally aligned with results of the whole-cohort analyses (**Supplementary Figures S3, S4**), distinct differences were seen in some subgroups. Interestingly, for patients with an immunosuppressive TME, represented by low CD8^+^ cell infiltration, high CD163^+^ cell infiltration or a low CD8^+^/CD163^+^ cell ratio, NAT with nCRT was associated with improved survival compared to nCT (**Figure 4A**). We explored these findings further by stratifying patients into long-term or short-term survivors, based on a 24-month cut-off, which showed additional evidence that a pro-tumour TME is associated with short-term survival for patients treated with nCT (**Figure 4B**). For MPR, we found that in patients treated with nCRT, tumour and stroma CD163^+^ cell densities were significantly higher in patients who achieved MPR, while no associations were observed in nCT patients (**Figure 4C**).

### 3.6 Comparison of the pre- and post-NAT immune landscape

**Figure 5:**
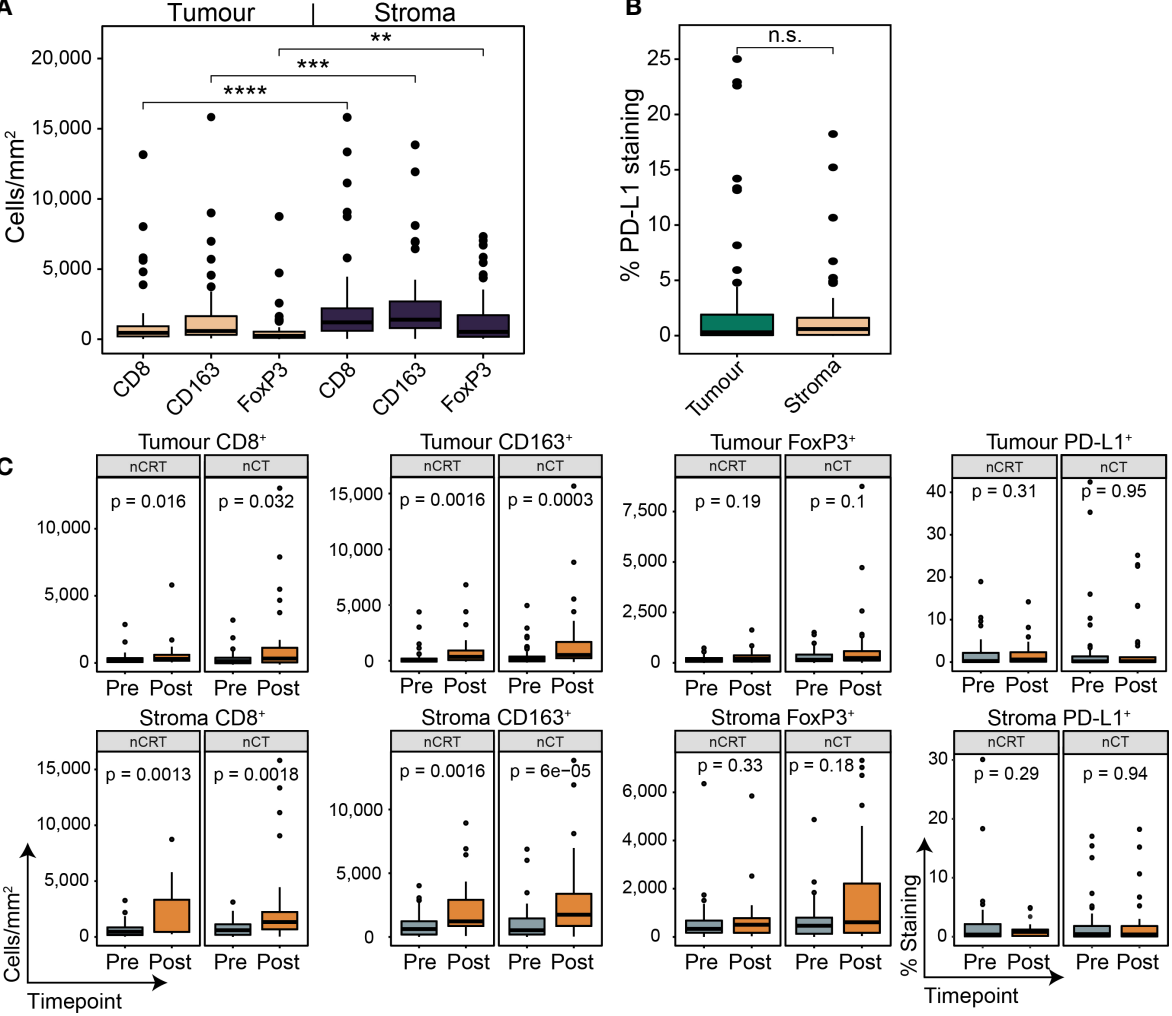
The spatial contexture of the tumour immune landscape following neoadjuvant therapy. Multiplex/single stain IHC from matched tumour containing post-NAT samples. In line with pre-NAT samples, higher densities of cell types was seen in stromal tissue compared to tumour **(A)**, with no difference seen for PD-L1 ^+^ expression **(B)**. PD-L1 staining is reported as the percentage area of marker activity for a total region. **(C)** When stratifying for treatment type, increases in tumoural and stromal CD8 ^+^ and CD163 ^+^ density was seen for nCT and nCRT, while no difference was seen for FoxP3^+^ density and PD-L1 ^+^ expression. Boxplots show the median (centre bar), the 1^st^ and 3^rd^ quartiles (upper and lower limit of boxes), and the largest and smallest value that is ≤ 1.5 times the interquartile range (whiskers). Comparisons were made using Wilcoxon rank-sum tests. P values are indicated or represented by: n.s., non-significant; **p <0.01; ***p <0.001; ****p <0.0001. nCRT, neoadjuvant CRT; nCT, neoadjuvant CT; n.s., non-significant.

We next assessed temporal changes in the TME under the effect of NAT using matched post-treatment samples. 63/107 (58.9%) of patients had a post-treatment sample which contained residual tumour; of which, 27/63 (42.9%) received nCRT, and 36/63 (57.1%) received nCT. Cell marker density and PD-L1 expression followed the same pattern as the pre-treatment samples (**Figures 5A, B**). In nCT and nCRT treated patients, CD8^+^ and CD163^+^ cell density was higher in the post-treatment samples in both tumour and stroma, while no difference was seen for FoxP3^+^ or PD-L1^+^ (**Figure 5C**). In post-NAT samples, no differences in cell density or PD-L1^+^ expression was detected between treatment groups (**Supplementary Figure S5**).

**Figure 6:**
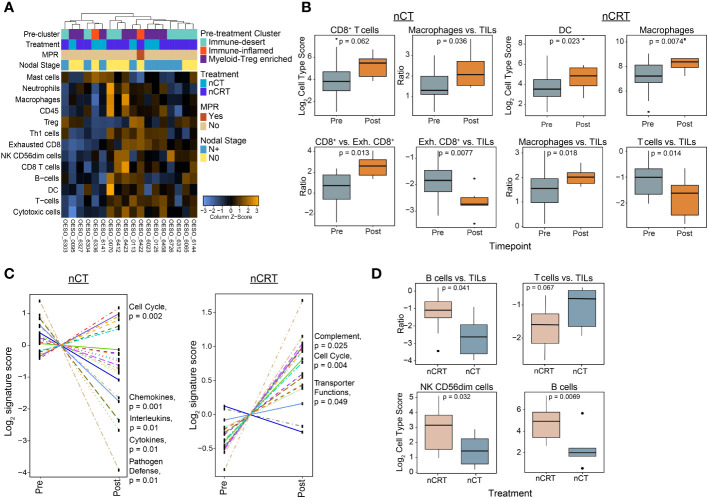
The post-treatment immune landscape of OAC using mRNA expression. NanoString mRNA expression profiling was used to profile the TME in 18 tumour-containing matched post-treatment OAC samples. **(A)** Unsupervised hierarchical clustering of NanoString-derived cell types. The top bars show the pre-treatment immune cluster and clinicopathological variables. **(B)** Cell score changes from pre to post therapy in chemotherapy and chemoradiotherapy treated patients. **(C)** Trend plots comparing gene expression pathway scores between pre and post-NAT. **(D)** Cell score differences between nCT and nCRT patients. Significant pathways are highlighted. Boxplots show the median (centre bar), the 1^st^ and 3^rd^ quartiles (upper and lower limit of boxes), and the largest and smallest value that is ≤ 1.5 times the interquartile range (whiskers). Comparisons were made using Wilcoxon rank-sum tests. P values are indicated in all plots. DC, dendritic cell; Exh, exhausted; NAT, neoadjuvant therapy; nCRT, neoadjuvant CRT; nCT, neoadjuvant CT; NK, natural killer; TIL, tumour infiltrating lymphocyte.

In the cohort of 44 patients used for pre-treatment NanoString mRNA profiling, 18/44 (40.9%) had post-treatment samples with residual tumour. 2/18 (11.1%) were from the pre-treatment immune-inflamed cluster, 8/18 (44.4%) from myeloid-Treg enriched, and 8/18 (44.4%) from immune-desert (**Figure 6A**). The majority (17/18) of patients were poor responders, with only one (OESO_6422) achieving MPR. Overall immune infiltration as represented by CD45 cells and total TILs was unchanged from pre to post therapy for both nCT and nCRT treated patients (**Supplementary Figures S6A, B**). Despite this, nCT treated patients showed a trend towards higher CD8^+^ cells post-therapy (p = 0.062), with less exhausted CD8^+^ T cells compared to CD8^+^ T cells and TILs post therapy (p = 0.013, and p = 0.0077, respectively; **Figure 6B**). nCRT patients had an increased macrophage (p = 0.0074) and dendritic cell signature (p = 0.023), as well as a decreased T cell to TIL ratio post treatment (p = 0.014; **Figure 6B**). Both nCT and nCRT treated patients showed increased macrophage numbers compared to TILs (p = 0.036, and p = 0.018, respectively; **Figure 6B**). Differential gene expression analyses revealed that, following nCRT, most genes were upregulated, with an admixture of pro- and anti-tumour genes reaching significance (**Supplementary Figure S6C**). Notably this included pro-tumour and negative immune regulatory genes such as *KIR inhibiting subgroup 2*, *CXCL12*, *CXCR4*, and *CD163*. nCT treated patients had more highly expressed genes pre-treatment, including immune-checkpoints *CD274*, and *IDO1*. The same pattern of regulation was seen for pathway signatures analysis, with nCT mostly down-regulating and nCRT mostly upregulating pathways (**Figure 6C**). Compared to nCRT, post-treatment samples following nCT showed lower total B cells (p = 0.0069), and B cells compared to TILs (p = 0.041; **Figure 6D**). nCT however, showed higher T cells compared to TILs and lower CD56^dim^ NK cells than nCRT patients post therapy (p = 0.067, and p = 0.032, respectively; **Figure 6D**). These findings indicate that NAT modalities result in the modulation of different immune signalling pathways, but overall decreased adaptive immune cell function, increased immunosuppressive myeloid functions and increased cell cycle signatures can be seen in treatment resistant tumours.

### 3.7 Functional responses to tumour antigens in OAC

**Figure 7:**
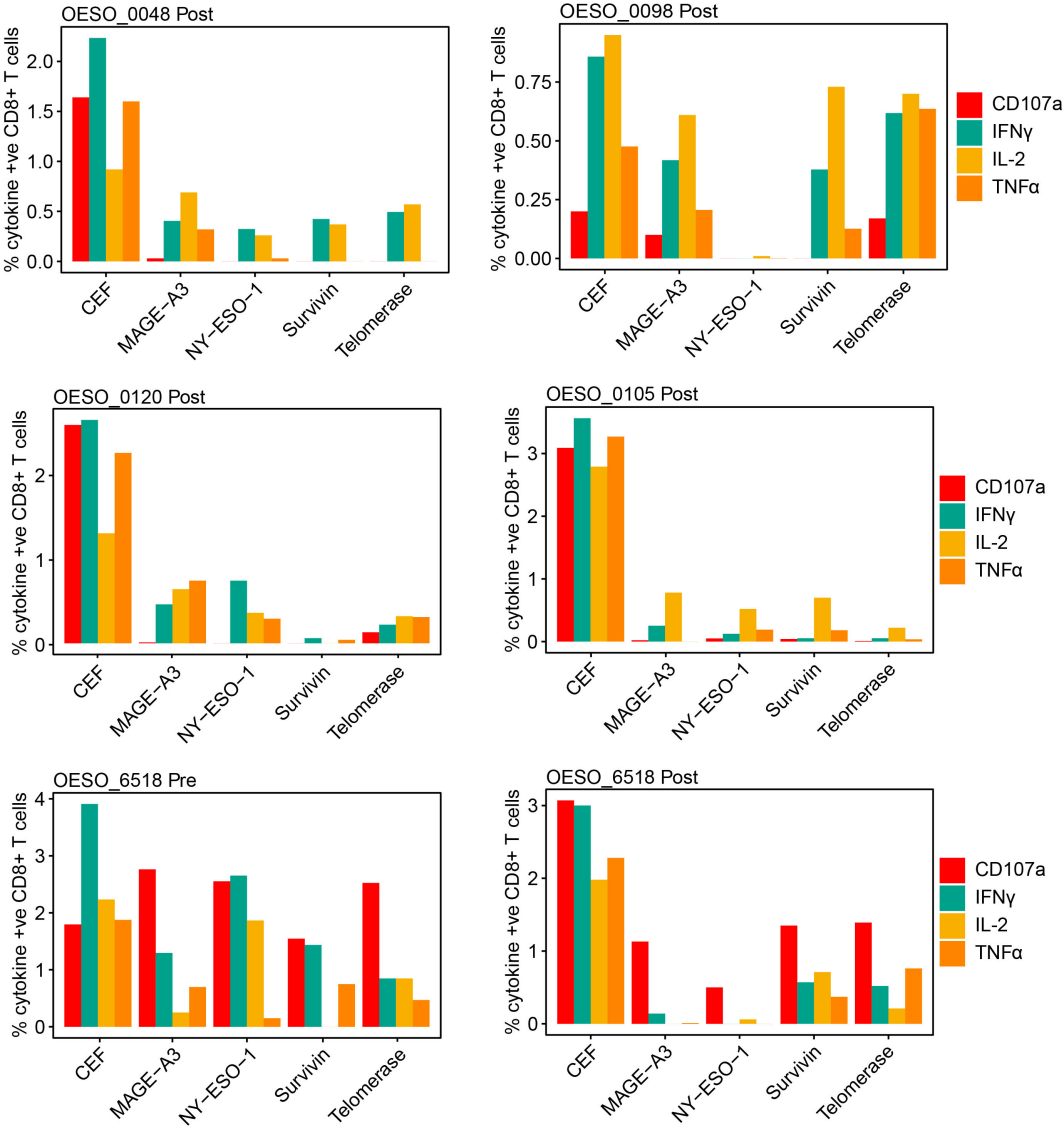
Functional cytokine responses to immunogenic tumour antigens. PBMCs from OAC patients were cultured in the presence of known immunogenic tumour antigens (NY-ESO-1, MAGE-A3, Survivin, Telomerase), or with a CEF pool or mock as control. Antigen specific functional responses were assessed using flow cytometry measurement of CD107a, IFNγ, IL-2, and TNFα release. The percentage of cytokine positive CD8^+^ cells are shown for each antigen in each patient following mock subtraction.

Finally, we sought to assess tumour antigen responses in OAC. Using PBMCs from HLA-A*02 patients, we assessed the presence of poly-functional CD8^+^ T cell responses to HLA-A*02 restricted tumour antigens (**Figure 7**). Prior to performing patient assays, HLA-A*02 healthy donor PBMCs were used to confirm the ability of the assay to produce cytokine responses to positive control stimuli (**Supplementary Figure S7**). This confirmed the ability of the assay to produce responses to both CytoStim™ and CEF. Due to being a more physiological stimulus, CEF was used in patient assays as a positive control. 5 HLA-A*02 patients had available PBMCs taken following NAT, 3 received nCRT (OESO_0048, OESO_0098, and OESO_0120), and 2 received nCT (OESO_0105, and OESO_6518). 1 patient (OESO_6518) had PBMCs from pre- and post-NAT. PBMCs from all patients showed detectable CD8^+^ T cell responses against the pool of viral antigens (CEF) used as a positive control, confirming the functional suitability of these cells (**Figure 7**). In nCRT patients, heterogeneous poly-functional responses to tumour antigens were identified in all 3 patients, although their extent was lower when compared to stimulation with the CEF peptide pool. In post-treatment PBMCs, only 1/2 nCT patients (OESO_6518) showed detectable tumour antigen specific CD8^+^ T cell responses, which again was attenuated compared to CEF. Of note, when we assessed pre-NAT PBMCs in OESO_6518, poly-functional cytokine responses were identified to multiple tumour antigens together with the expression of the CD107a degranulation marker indicating cytotoxic activity.

OESO_0120 had low intra-tumoural CD8^+^ cells in both pre- and post-tumour tissues, along with low intra-tumoural CD163^+^ and FoxP3^+^ cells in pre- tumour tissue, consistent with a likely immune desert phenotype. OESO_0105 which displayed only IL-2 responses to tumour antigens, had high intra-tumoural CD8^+^ cells in pre-NAT samples, but low CD8^+^ infiltration following therapy. Interestingly, OESO_0105, OESO_0120 and OESO_6518 were non-MPRs, raising the possibility that reduced or dysfunctional T cells responses to tumour antigens or low CD8^+^ T cell infiltration may be associated with failure to respond to NAT in OAC; however, this requires further testing in a larger cohort of samples.

## 4 Discussion

To improve outcomes for OAC patients, rational personalisation of therapeutic approaches is necessary. Biomarkers based on the underlying biological features of the tumour and its TME could be potential indicators of the likelihood of treatment response and patient survival and must account for the underlying vulnerabilities and resistance mechanisms that can affect treatment efficacy. It is well documented that immune-inflamed tumours have better clinical outcomes when treated with both standard therapies and immunotherapy compared to immune-altered (excluded or immunosuppressed) or immune-cold phenotypes (38). However, to date, only limited studies have investigated these critical biomarkers of therapy response in OAC. Using mRNA expression profiling, we found a subgroup of OAC patients with an immune-inflamed TME, replete with a functional adaptive immune response that was associated with improved clinicopathological outcomes and survival. The worst survival was seen for patients with a myeloid-Treg enriched TME. The influence of myeloid cell populations and Tregs on immunosuppression and poor clinicopathological outcomes are documented in OAC and other cancers, and function via various tumour and immune cell mechanisms (16, 17, 20). Recent evidence has shown that in other cancer types ferroptotic neutrophils can promote immunosuppression via T cell inhibition (39), while MYC pathway activation, which was found in T cell depleted OACs has been shown to promote macrophage and neutrophil infiltration, with loss of T cell, B cell, and NK cell infiltration (16). We also identified an immune-desert cluster that showed the lowest rate of MPR, but not the worst survival. Interestingly, this cluster also had a significantly higher rate of NAT with nCRT. Overall, these findings suggest that, in the absence of an inflamed TME, the balance of anti- and pro-tumour immune cells within the TME impacts the quality of functional immune responses and may influence patient outcomes. Furthermore, regardless of the NAT regimen used, patients with an underlying anti-tumour immune response appear more likely to achieve treatment response and long-term survival.

Using the underlying principles of the immune contexture, we aimed to determine the relevance of spatial immune cell distribution in patient outcomes. We found that a high density of CD8^+^ T cells both within and surrounding tumours was associated with improved survival, which is in line with previous studies (27, 30, 40). In addition, high CD163^+^ cell density was associated with shorter survival, also supporting findings of previous studies (18, 26, 28). FoxP3^+^ Treg cell density had no influence on outcomes in our cohort. Of note, the prognostic relevance of FoxP3^+^ cells shows contrasting results in the literature (19, 20, 27, 41), suggesting that the role FoxP3^+^ cells play within the TME needs to be considered in the context of other stromal or infiltrating immune cells and their functional interactions. Supporting our mRNA expression findings, high anti-tumour/pro-tumour immune cell ratios were associated with favourable survival outcomes. Two recent studies have demonstrated that a high CD8^+^/CD163^+^ cell ratio was associated with treatment response in OAC patients (17, 19). Interestingly, neither of these studies demonstrated a survival benefit, likely due to their limited sample sizes. When assessing TC and TM immune cell location, we found that CD8^+^ cell location reflected our findings of overall intra-tumoural immune cell infiltration, with improved survival in high TC and TM patients. For FoxP3^+^ cells, in contrast to overall intra-tumoural infiltration, we found that high TC infiltration was associated with improved OS, especially in the setting of high TC CD8^+^ infiltration. This may reflect the homeostatic role of Tregs which accompany effector T cells in the setting of an active anti-tumour immune response (12), however could also be due to FoxP3 expression as a result of T cell activation (42, 43).Of note, high TM, but not TC, CD163^+^ cell infiltration was associated with poor survival, which was evident even in the presence of high TM CD8^+^ cell infiltration. These findings are similar to those of a previous study (18), however the authors also demonstrated poor survival for cases showing high TC M2-macrophage infiltration. M2-macrophages have been shown to promote tumour development through multiple mechanisms (44). In the tumour periphery, M2-macrophages can exert inhibitory effects on CD8^+^ T cells and the broader anti-tumour immune responses, impairing tumour cell killing and negatively impacting survival (45). As discussed, OAC has been shown to have a largely immune-excluded/immunosuppressed phenotype (16, 17), and the additional analyses reported here support these findings with higher densities of all immune cells seen in stroma compared to tumour in both pre- and post-treatment samples. Immune-excluded tumours are associated with impaired outcomes, and although the host immune system can mount T-cell mediated responses, these cells are unable to penetrate the tumour, effects that our results suggest are driven in part by the presence of M2-macrophages at tumour margins. A potential confounder with these results is lack of standard definition for a tumour core and margin zone, and the heterogeneity in tumour area between samples. This heterogeneity may result in inter-sample differences in the area of measured tumour core between samples, thus these findings, although encouraging, require further confirmation in larger data sets to prove their clinical significance.

In OAC, nCRT results in higher rates of MPR, but not OS Given the significant difference in MPR rates but not survival between nCRT and nCT, we stratified patients by treatment modality and performed exploratory analyses to determine how the pre-treatment immune landscape impacts treatment response and outcomes. Previous reports have indicated that, in addition to directly inducing cancer cell death, RT may invoke immunogenic cell death and promote an *in situ* tumour vaccination effect, resulting in immune-mediated tumour clearance (24, 46). In our study, the addition of RT improved survival for patients with evidence of an immunosuppressive TME prior to therapy. In patients with high CD163^+^ cell infiltration, treatment with nCT resulted in a significantly worse survival compared to patients treated with nCRT. This was supported by the presence of high CD163^+^ cell infiltration and related functional responses in pre-treatment biopsies of nCRT-treated patients who achieved MPR. Overall, these findings suggest that RT may ameliorate the effects of an underlying immunosuppressive TME compared to chemotherapy. Mechanisms underpinning the observed clinical benefits for RT compared to chemotherapy in the setting of high CD163^+^ cell infiltration are, however, difficult to decipher. This is due to the high phenotypic plasticity of TAMs and their inherent radioresistance (47, 48). In preclinical settings, RT has been shown to re-program the pro-tumour TAM phenotype towards the anti-tumour M1-phenotype when administered at low- to moderate-dose regimens (48). However, RT dosing for clinical treatment of OAC uses high-dose regimens, which is reported to induce M2-polarisation in multiple cancer types (48). The pathways and external factors involved in TAM polarisation are highly complex, and to date are incompletely understood, especially in the context of the anti-tumour immunogenic effects of RT. More research is required to determine both the biological effects mediated by TAM-infiltration on the TME, as well as the mechanistic effects of RT and chemotherapy on TAM phenotypes in OAC (24, 46–48).

We next focused on the post-treatment immune landscape. When stratified for treatment type, both nCT and nCRT groups resulted in higher CD8^+^ and CD163^+^ cell density in tumour and stroma, with no difference seen for FoxP3^+^ density and PD-L1^+^ expression. Two previous studies have investigated changes in cell density following CRT in OAC with conflicting results; one found an increase in CD8^+^ cell density and no change in FoxP3^+^ cell density (27), while another reported significantly lower FoxP3^+^ and CD163^+^ cell density, and no change in CD8^+^ cell density (19). For nCT patients, a previous study also reported increased intra-tumour CD8^+^ T cell density, but decreased FoxP3^+^ cell density post therapy (29). Direct comparisons with other studies is somewhat limited however by the observed heterogeneity in patient characteristics and treatment regimens. Furthermore, a lack of uniform cell markers and need for multiple markers for accurate cell representation is also a limitation that can lead to divergent results between studies. Post-NAT mRNA expression data supported our mIF findings, showing an increase in the proportion of macrophages in both nCT and nCRT patients and increases in CD8^+^ cells post-nCT. Post-nCRT patients displayed lower T cells vs. total TILs compared to both pre-NAT samples and post-nCT samples, possibly reflecting the radiosensitivity of T cells. Differential gene expression and functional pathway comparison from pre- to post-therapy, showed general upregulation of immune genes and most pathways in nCRT patients, with the opposite effect seen in nCT patients, giving further insight into the immunomodulatory effects of RT in OAC. For nCT patients, the significant upregulation of immune-checkpoint targets in the pre-NAT TME provides the rationale for nCT + ICB combination therapies in these patients, and results of the KEYNOTE-585 and MATTERHORN trials assessing these combinations are anticipated (49, 50).

It is worth noting that only post-NAT samples with residual tumour were assessed. It is plausible that a treatment resistance mechanism in these tumours stems from poor tumour immunogenicity, and immune-resistance, which may be due to immune escape from mechanisms such as immunoediting (51, 52), or allele-specific HLA loss (53). Surprisingly, no difference was seen in post-NAT immune cell densities between NAT-types suggesting potential common mechanisms of immune-resistance in these tumours, an avenue that requires further exploration. Understanding the TME of tumours post-NAT is clinically relevant given the results of the CheckMate-577 trial where patients with residual tumour were given Nivolumab following nCRT and surgery (10). In CheckMate-577, disease-free survival (DFS) was significantly prolonged in a subset of OAC patients. One potential area of interest underpinning these results is the role of B cells and tertiary lymphoid structures (TLSs) in treatment response and outcome. In our study, post-CRT patients displayed higher B cell signatures compared to pre-treatment. A recent study using IHC identified the presence of B cells and TLSs in both the pre- and post-treatment TME of OAC tumours (54). In contrast to our findings, however, both were decreased following therapy. We found that *TNFSF13B*, which encodes for B cell activating factor (BAFF) was upregulated in the pre-treatment TME of MPR patients. BAFF enhances B cell functions and has been shown to attenuate immunosuppressive myeloid cell infiltration and PD-L1 expression in melanoma (55). Furthermore BAFF has also been shown to augment the antitumor immune response in melanoma via T cell activation, T helper 1 cell polarisation, and promotion of a memory phenotype, with patients with high BAFF expression showing improved OS (56). B cells and TLSs have been shown to be prognostically significant in patients treated with immunotherapy in other cancers (57), and thus further investigation of the roles of B cells and TLSs in OAC may inform both patient selection, and timing of ICB in future OAC trials.

Finally, we assessed for the presence of peripheral circulating tumour antigen-specific T cells. Detection of responses to known tumour antigens provides evidence for efficient tumour immunogenicity due to efficient tumour presentation and T cell recognition of these antigens. Our results suggest patients who achieved MPR had tumour-specific circulating T cells showing overall better cytokine responses than non-MPR patients. Although the level of activation seen for these responses were low compared to CEF, this result is not unexpected as pre-expansion of antigen specific T cells wasn’t performed. Given this, one limitation of our study is the low PBMC response seen to CEF stimulation, which may be improved in future experiments through assay modification. We also note that heterogeneity was seen among treatment modality, response rates, and timepoint, highlighting the complexities of the tumour antigen presentation pathways by tumours and the associated immune cell responses. Our study is limited in that only a limited number of samples as well as a limited number of PBMCs from HLA-A*02 patients were available. This precluded statistical comparison and indicates that a larger series should be investigated to more conclusively highlight clinically relevant correlations.

In summary, with the current one-size-fits-all approach a plateau in treatment effect has occurred, and personalisation of therapy is required to improve outcomes. Here, we have shown that in patients with an immune-inflamed TME, treatment with conventional nCT or nCRT is associated with favourable outcomes. However, in OAC patients with immunosuppressed or excluded TMEs, which appears to be driven by CD163^+^ cells, treatment with nCRT appears to confer benefit. Extrapolation of our data to results of the CheckMate-577 trial, which essentially assessed ICB in nCRT non-responders suggests that immunosuppressive changes in the TME can be ameliorated with ICB to improve DFS in a subset of patients. However, the degree of treatment response required to achieve clinical benefit from ICB is currently unknown, and future translational studies are required to determine which patients will benefit. Overall, our results indicate that assessment of the pre-treatment TME in OAC patients may identify those who will response best to different NAT regimens.

## Data availability statement

The original contributions presented in the study are included in the article/**Supplementary Material**, further inquiries can be directed to the corresponding author/s.

## Ethics statement

The studies involving humans were approved by Metro South Health Research Ethics Committee, The University of Queensland Ethics Committee, and the QIMR Berghofer Human Research Ethics Committee. The studies were conducted in accordance with the local legislation and institutional requirements. The participants provided their written informed consent to participate in this study.

## Author contributions

Study design: JL, NW, RD, and AB. Sample Collection and processing: AB, VB, LA, KP, and AGITG DOCTOR Investigators. Experimental procedures and data analyses: JL, VB, LA, IB, SS, GL, VA, LK, and SW. Original draft: JL, RD, and AB. Review and editing: all authors. All authors contributed to the article and approved the submitted version.

## Group member of AGITG DOCTOR Investigators

Trial Management Committee:

Andrew Barbour, John Simes, Euan Walpole, Gang Tao Mai, David Watson, Chris Karapetis, Val Gebski, Liz Barnes, Martijn Oostendorp, Kate Wilson

National Health and Medical Research Council Clinical Trial Centre, Sydney, NSW, Australia:

John Simes, Val Gebski, Louse Barnes, Martijn Oostendorp, Kate Wilson

Calvary Mater Hospital, Newcastle, NSW, Australia:

Stephen Ackland

Nepean Hospital, Sydney, NSW, Australia:

Jenny Shannon

Sydney Adventist Hospital, Sydney, NSW, Australia:

Gavin Marx

Royal Brisbane and Women’s Hospital, Brisbane, QLD, Australia:

Matthew Burge, Robert Finch

Princess Alexandra Hospital, Brisbane, QLD, Australia:

Andrew Barbour, Euan Walpole, Janine Thomas

Townsville Hospital, Townsville, QLD, Australia:

Suresh Varma

Flinders Medical Centre, Adelaide, SA, Australia:

Chris Karapetis, David Watson

Royal Hobart Hospital, Hobart, TAS, Australia:

Louise Nott
