# Supplementary material for: The oesophageal adenocarcinoma tumour immune microenvironment dictates outcomes with different modalities of neoadjuvant therapy – results from the AGITG DOCTOR trial and the cancer evolution biobank
Source: Front Immunol. 2023 Oct 12;14:1220129. doi: 10.3389/fimmu.2023.1220129 (PMC10642165; doi:10.3389/fimmu.2023.1220129)
Supplement: Supplementary file 1 [file Presentation_1.pptx]

## Slide 1
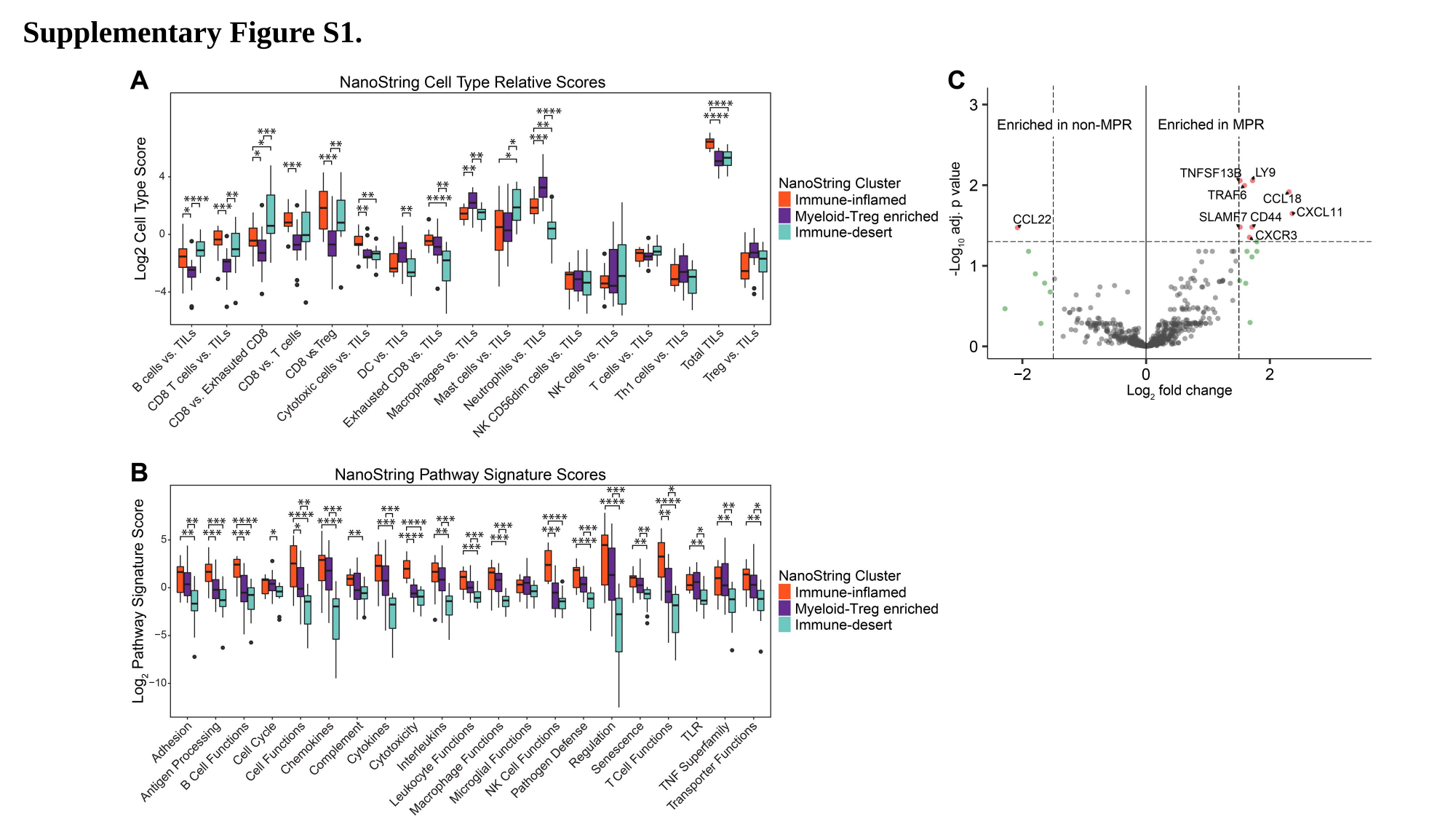

# Supplementary Figure S1.

## Slide 2
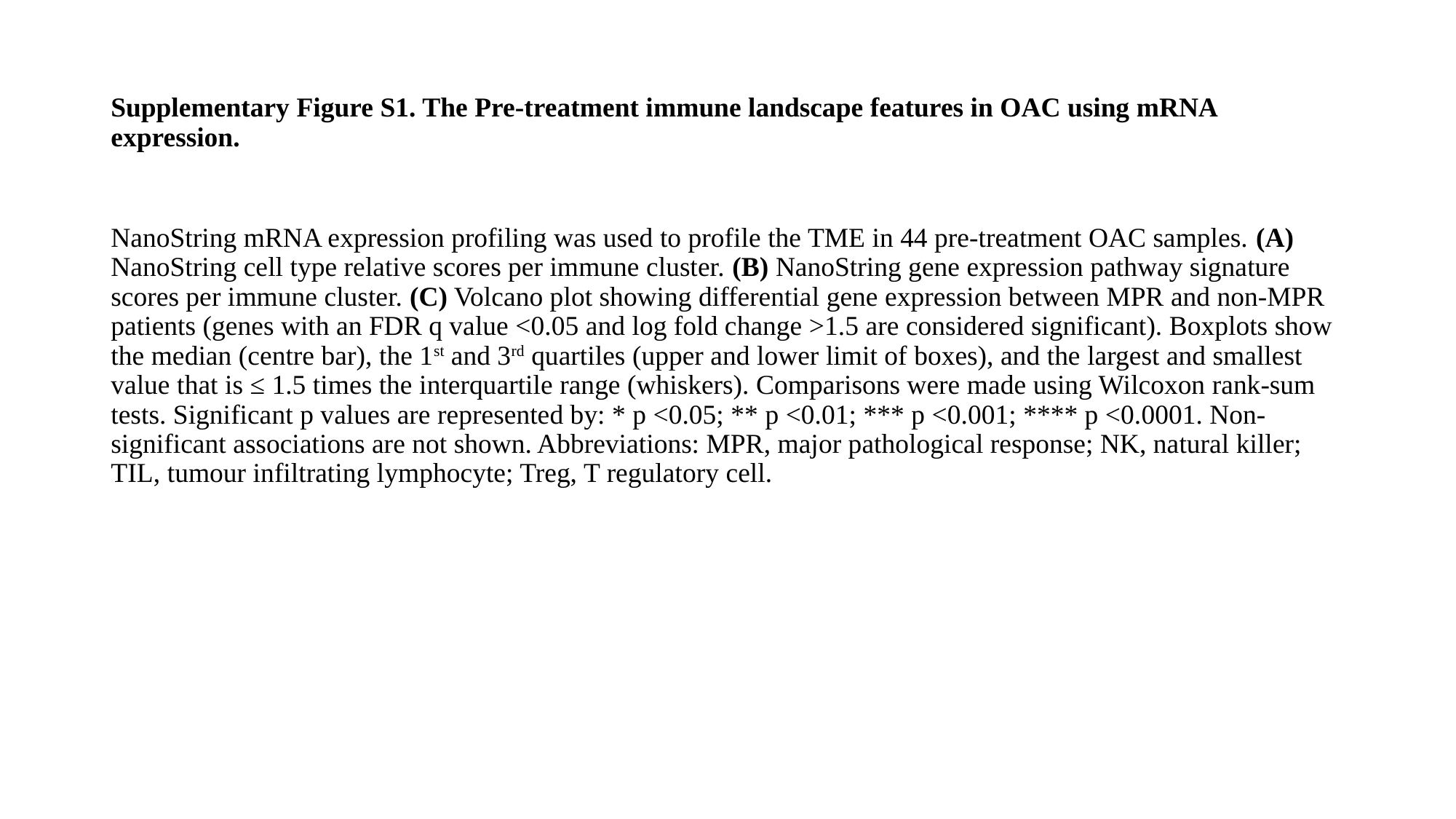

# Supplementary Figure S1. The Pre-treatment immune landscape features in OAC using mRNA expression.
NanoString mRNA expression profiling was used to profile the TME in 44 pre-treatment OAC samples. (A) NanoString cell type relative scores per immune cluster. (B) NanoString gene expression pathway signature scores per immune cluster. (C) Volcano plot showing differential gene expression between MPR and non-MPR patients (genes with an FDR q value <0.05 and log fold change >1.5 are considered significant). Boxplots show the median (centre bar), the 1st and 3rd quartiles (upper and lower limit of boxes), and the largest and smallest value that is ≤ 1.5 times the interquartile range (whiskers). Comparisons were made using Wilcoxon rank-sum tests. Significant p values are represented by: * p <0.05; ** p <0.01; *** p <0.001; **** p <0.0001. Non-significant associations are not shown. Abbreviations: MPR, major pathological response; NK, natural killer; TIL, tumour infiltrating lymphocyte; Treg, T regulatory cell.

## Slide 3
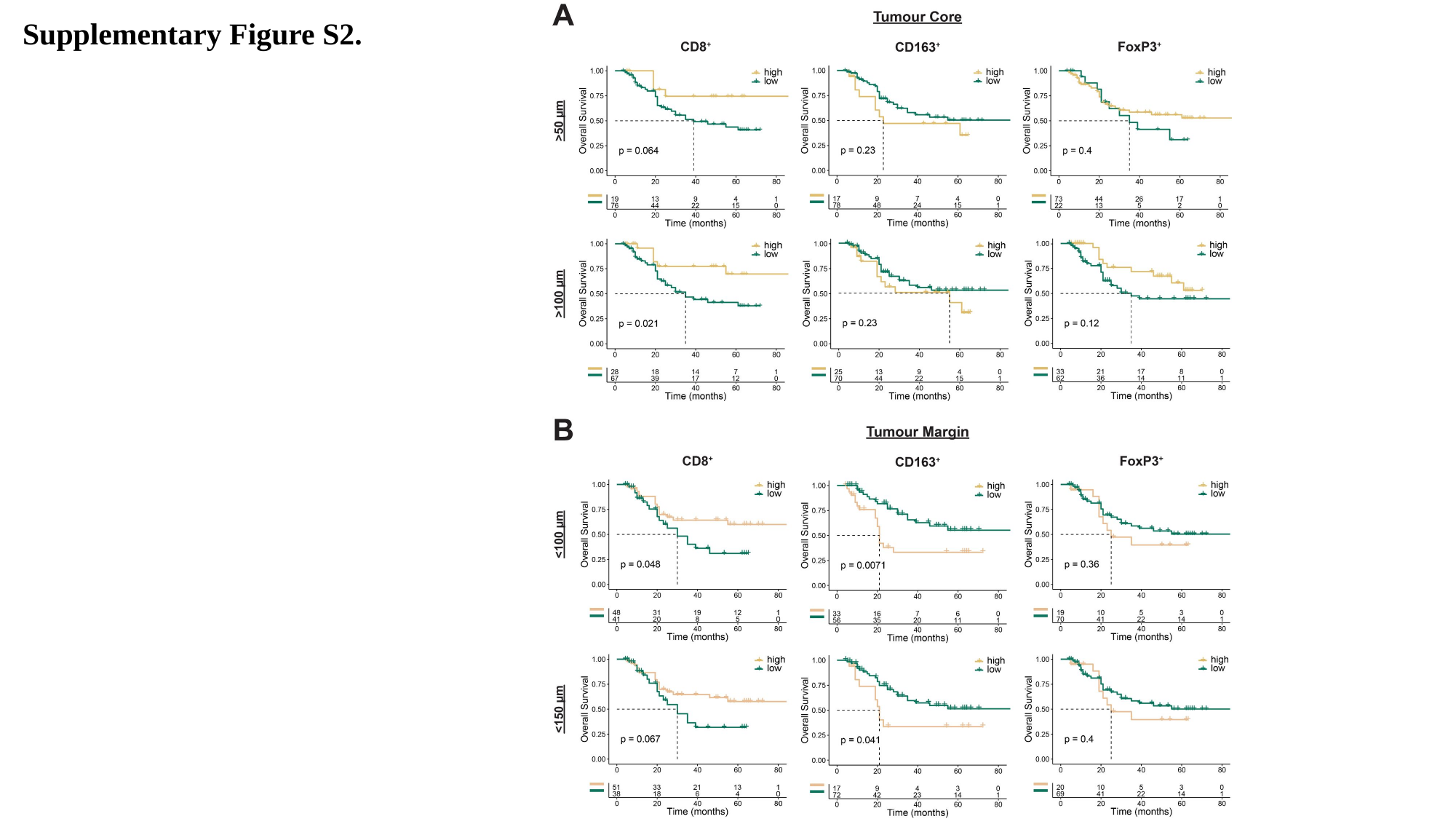

Supplementary Figure S2.

## Slide 4
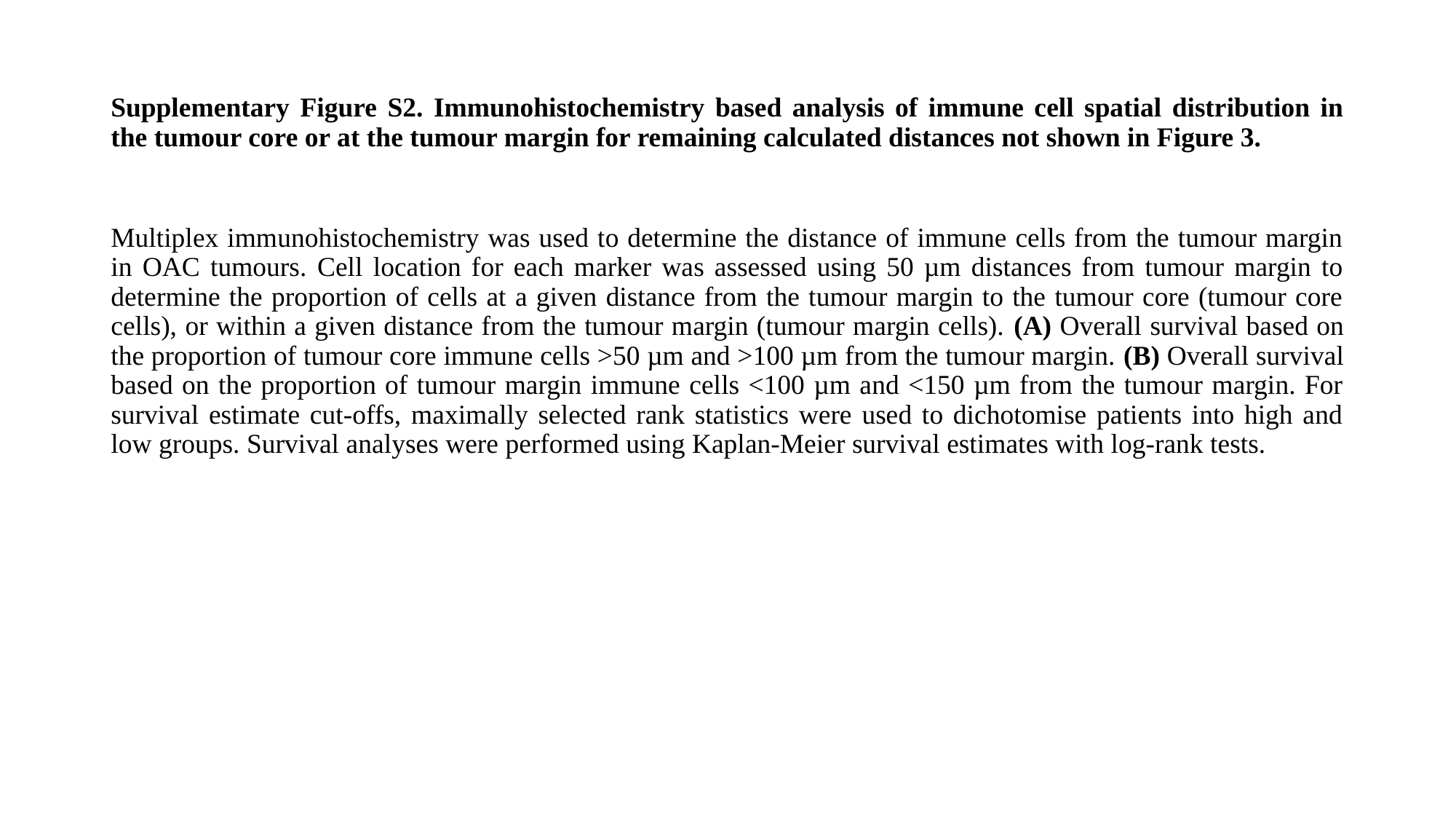

# Supplementary Figure S2. Immunohistochemistry based analysis of immune cell spatial distribution in the tumour core or at the tumour margin for remaining calculated distances not shown in Figure 3.
Multiplex immunohistochemistry was used to determine the distance of immune cells from the tumour margin in OAC tumours. Cell location for each marker was assessed using 50 µm distances from tumour margin to determine the proportion of cells at a given distance from the tumour margin to the tumour core (tumour core cells), or within a given distance from the tumour margin (tumour margin cells). (A) Overall survival based on the proportion of tumour core immune cells >50 µm and >100 µm from the tumour margin. (B) Overall survival based on the proportion of tumour margin immune cells <100 µm and <150 µm from the tumour margin. For survival estimate cut-offs, maximally selected rank statistics were used to dichotomise patients into high and low groups. Survival analyses were performed using Kaplan-Meier survival estimates with log-rank tests.

## Slide 5
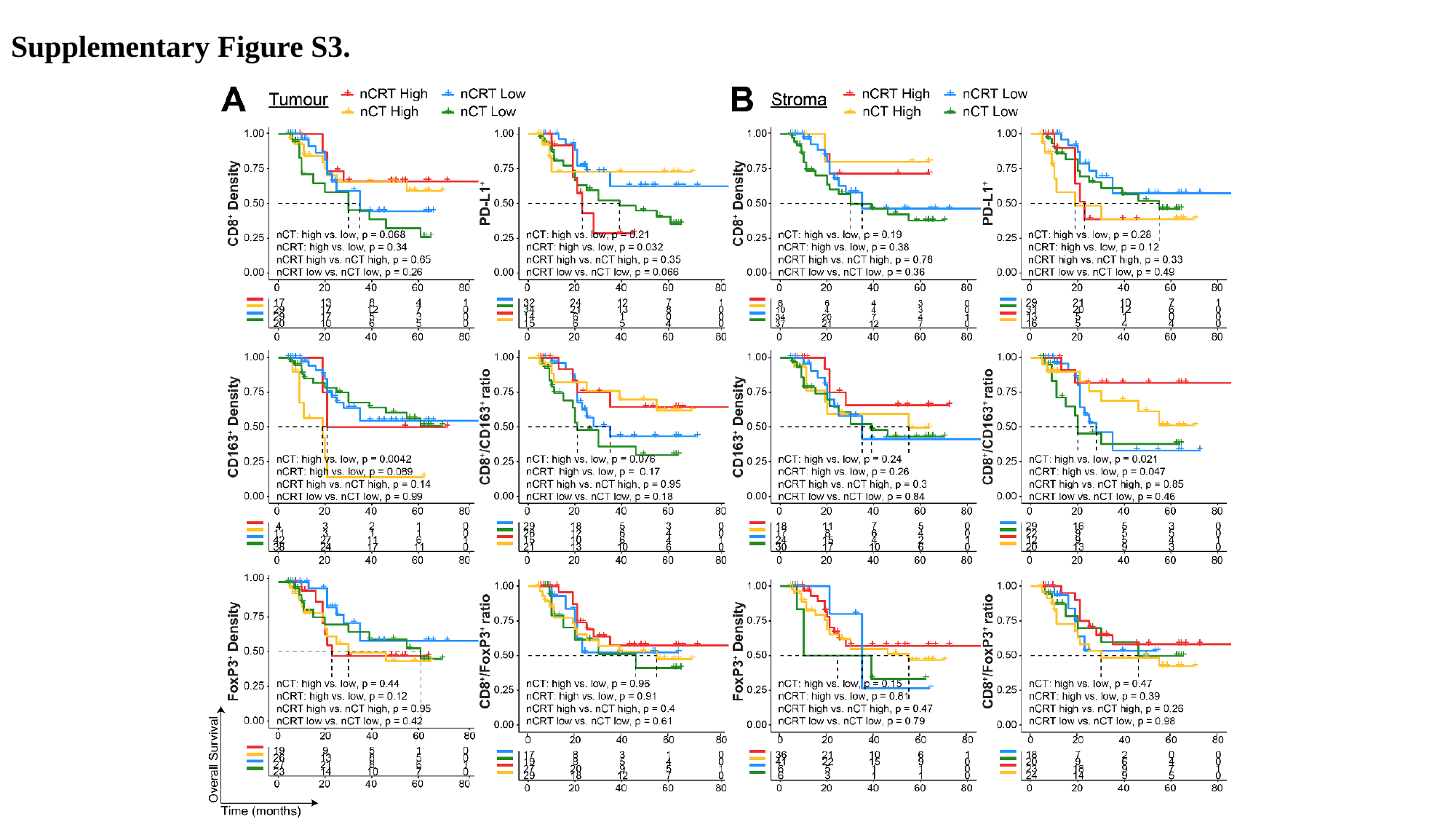

# Supplementary Figure S3.

## Slide 6
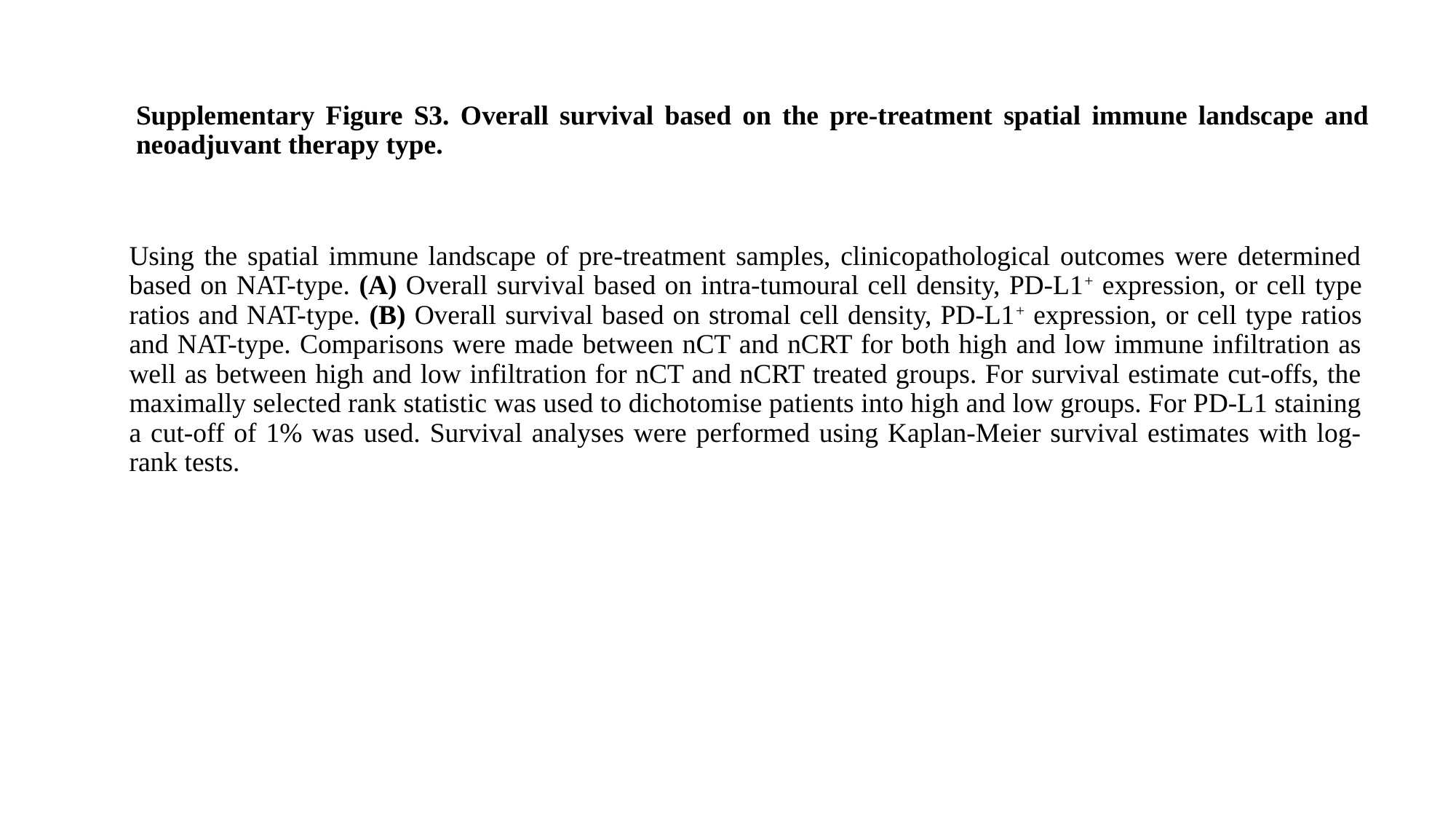

Supplementary Figure S3. Overall survival based on the pre-treatment spatial immune landscape and neoadjuvant therapy type.
Using the spatial immune landscape of pre-treatment samples, clinicopathological outcomes were determined based on NAT-type. (A) Overall survival based on intra-tumoural cell density, PD-L1+ expression, or cell type ratios and NAT-type. (B) Overall survival based on stromal cell density, PD-L1+ expression, or cell type ratios and NAT-type. Comparisons were made between nCT and nCRT for both high and low immune infiltration as well as between high and low infiltration for nCT and nCRT treated groups. For survival estimate cut-offs, the maximally selected rank statistic was used to dichotomise patients into high and low groups. For PD-L1 staining a cut-off of 1% was used. Survival analyses were performed using Kaplan-Meier survival estimates with log-rank tests.

## Slide 7
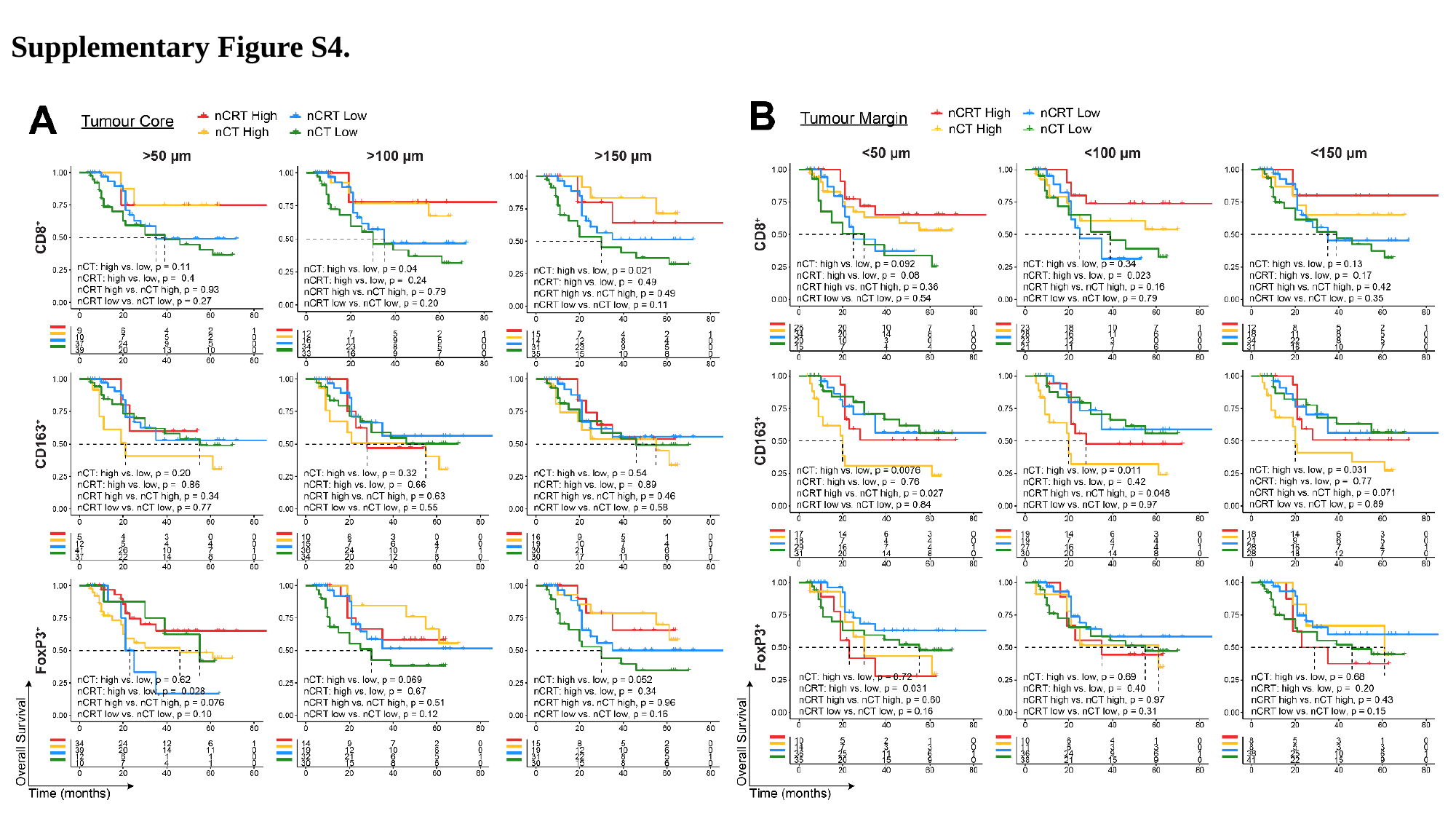

# Supplementary Figure S4.

## Slide 8
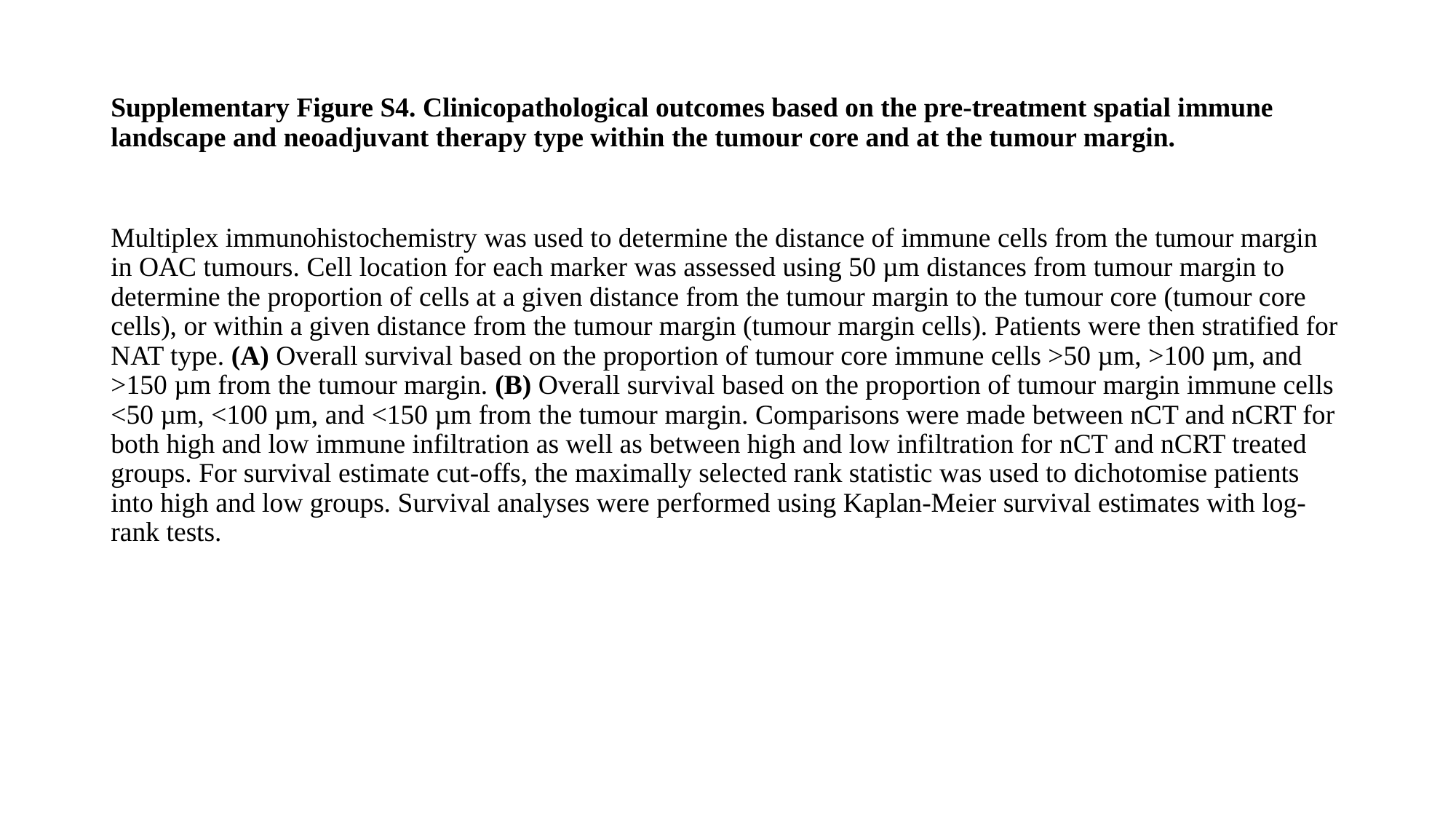

# Supplementary Figure S4. Clinicopathological outcomes based on the pre-treatment spatial immune landscape and neoadjuvant therapy type within the tumour core and at the tumour margin.
Multiplex immunohistochemistry was used to determine the distance of immune cells from the tumour margin in OAC tumours. Cell location for each marker was assessed using 50 µm distances from tumour margin to determine the proportion of cells at a given distance from the tumour margin to the tumour core (tumour core cells), or within a given distance from the tumour margin (tumour margin cells). Patients were then stratified for NAT type. (A) Overall survival based on the proportion of tumour core immune cells >50 µm, >100 µm, and >150 µm from the tumour margin. (B) Overall survival based on the proportion of tumour margin immune cells <50 µm, <100 µm, and <150 µm from the tumour margin. Comparisons were made between nCT and nCRT for both high and low immune infiltration as well as between high and low infiltration for nCT and nCRT treated groups. For survival estimate cut-offs, the maximally selected rank statistic was used to dichotomise patients into high and low groups. Survival analyses were performed using Kaplan-Meier survival estimates with log-rank tests.

## Slide 9
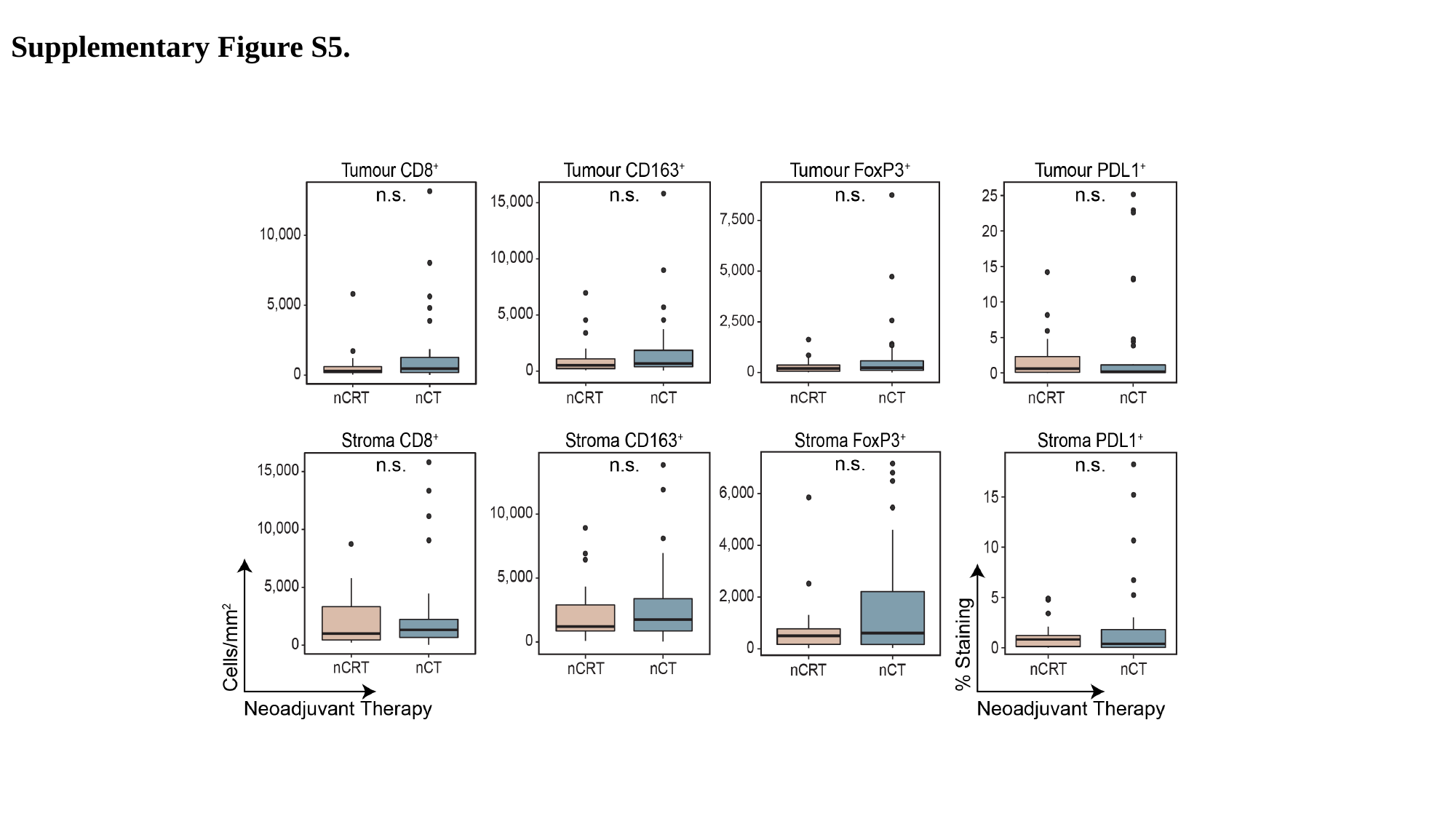

# Supplementary Figure S5.

## Slide 10
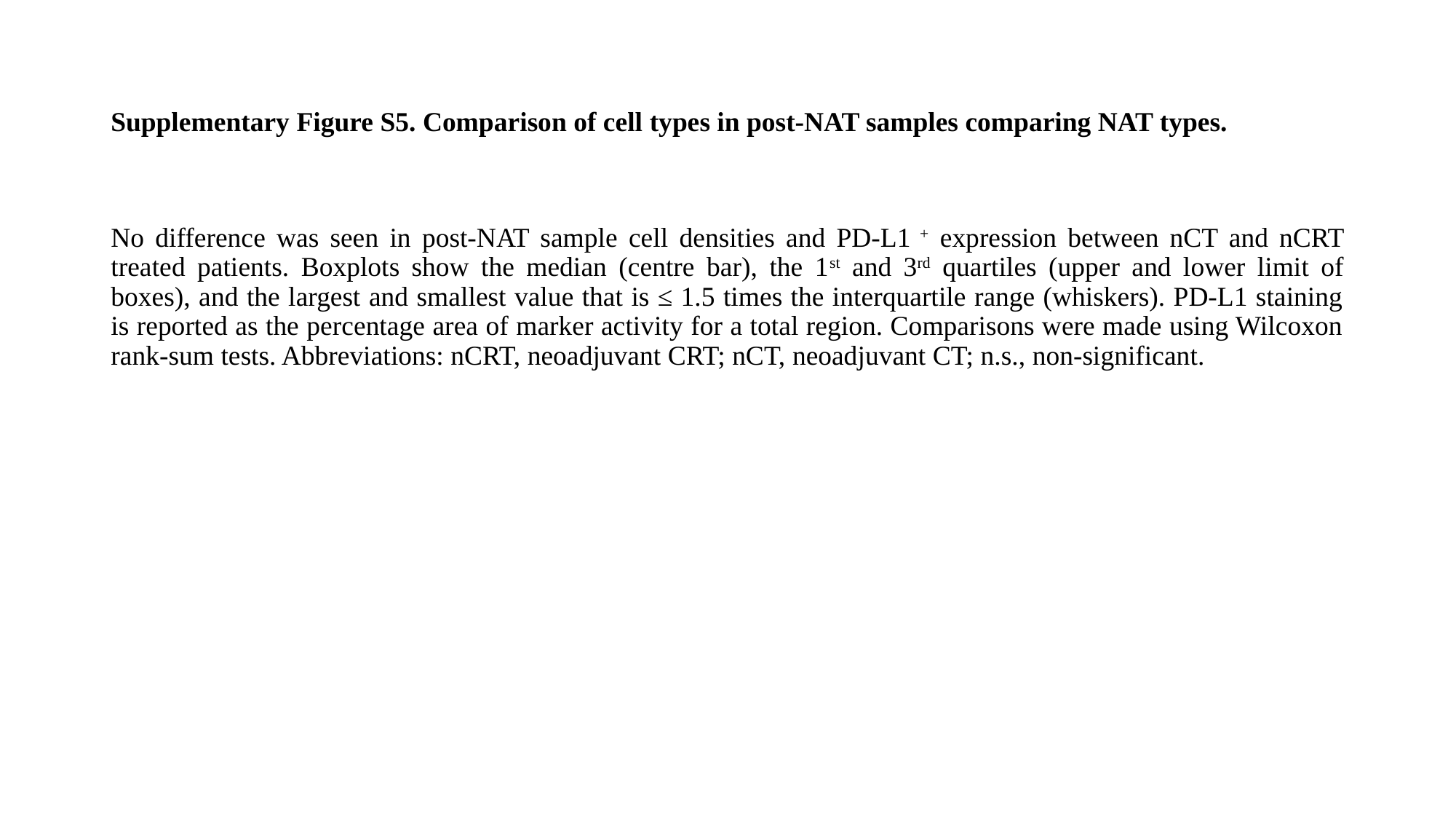

# Supplementary Figure S5. Comparison of cell types in post-NAT samples comparing NAT types.
No difference was seen in post-NAT sample cell densities and PD-L1 + expression between nCT and nCRT treated patients. Boxplots show the median (centre bar), the 1st and 3rd quartiles (upper and lower limit of boxes), and the largest and smallest value that is ≤ 1.5 times the interquartile range (whiskers). PD-L1 staining is reported as the percentage area of marker activity for a total region. Comparisons were made using Wilcoxon rank-sum tests. Abbreviations: nCRT, neoadjuvant CRT; nCT, neoadjuvant CT; n.s., non-significant.

## Slide 11
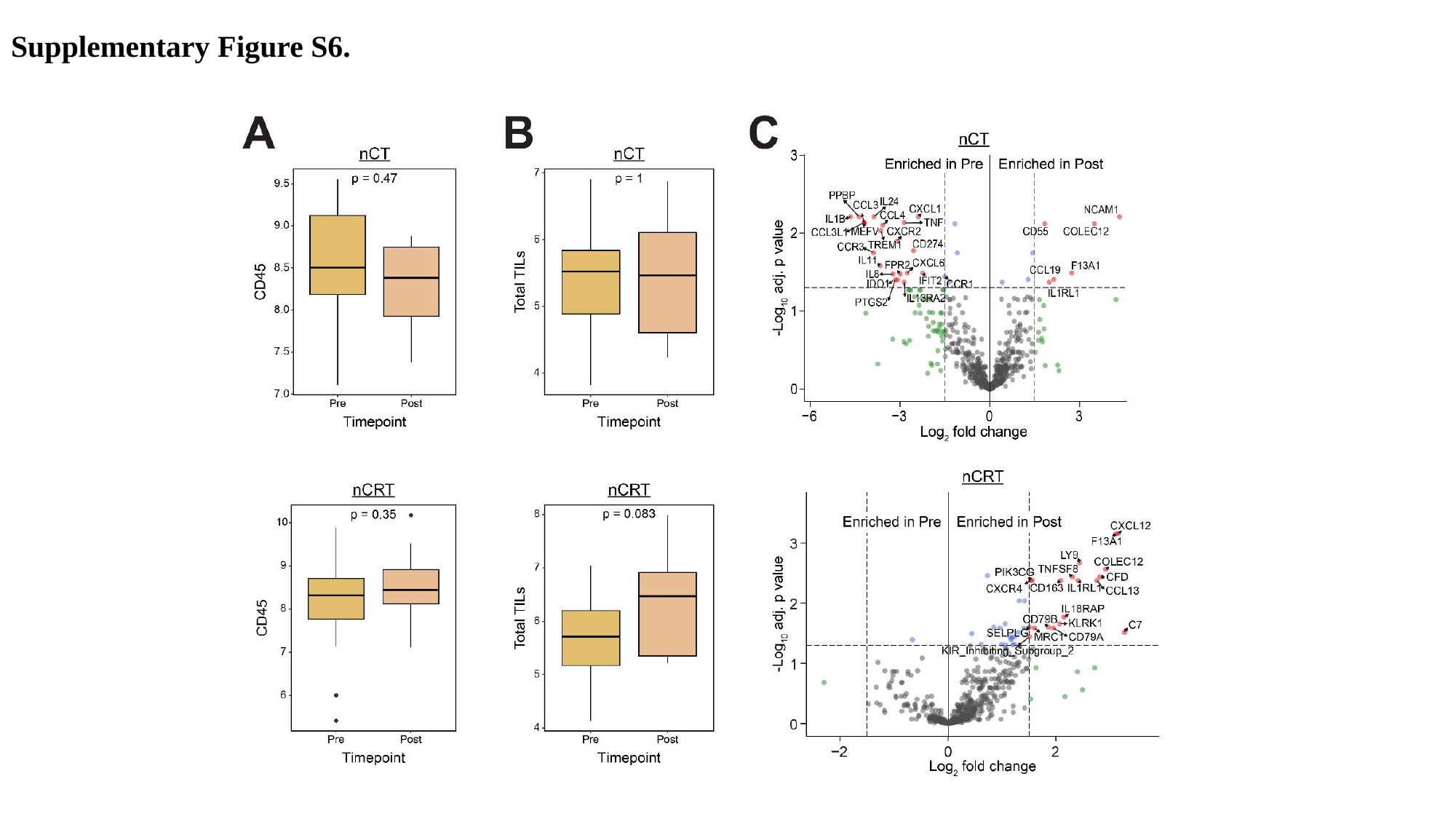

# Supplementary Figure S6.

## Slide 12
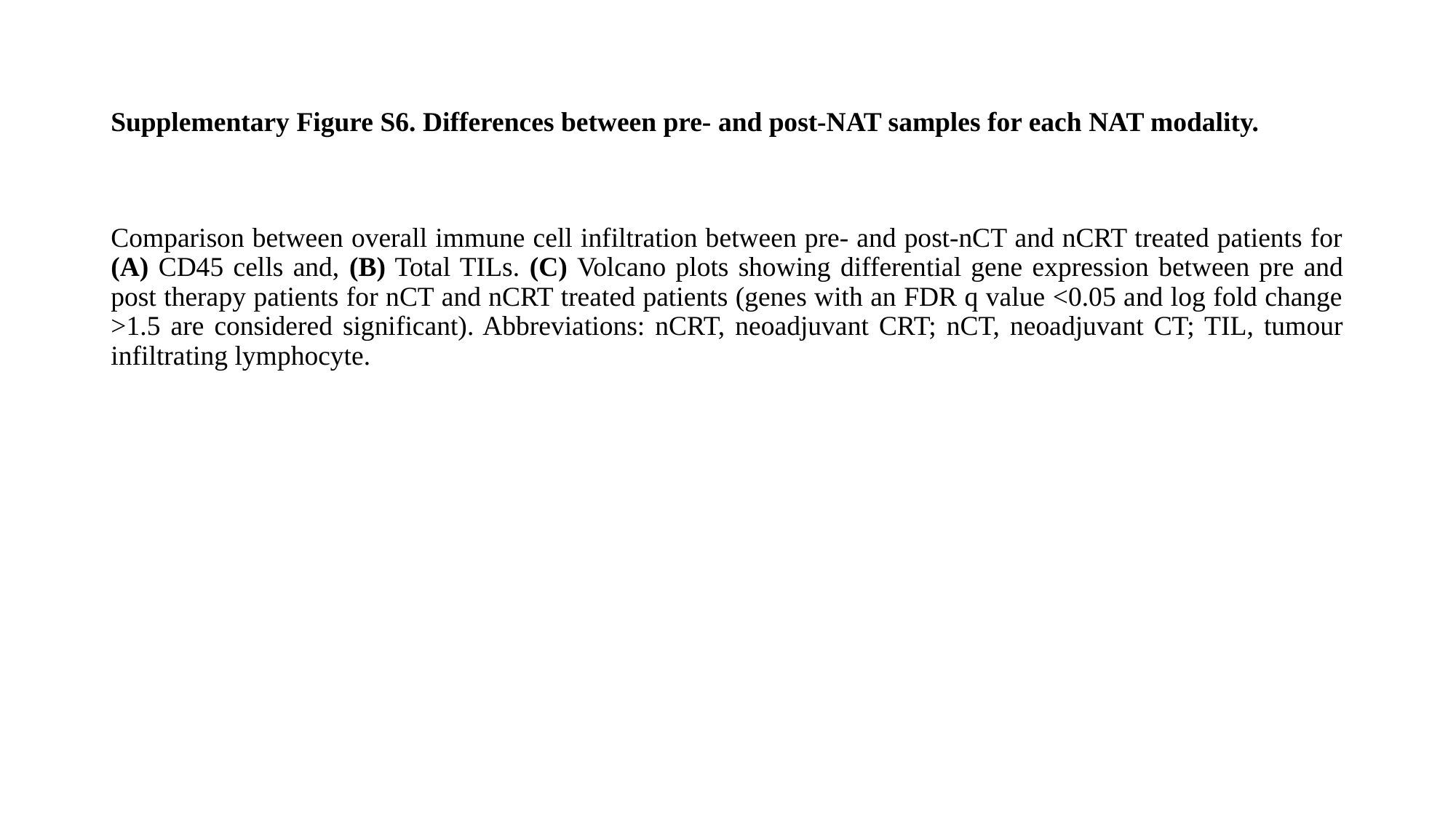

# Supplementary Figure S6. Differences between pre- and post-NAT samples for each NAT modality.
Comparison between overall immune cell infiltration between pre- and post-nCT and nCRT treated patients for (A) CD45 cells and, (B) Total TILs. (C) Volcano plots showing differential gene expression between pre and post therapy patients for nCT and nCRT treated patients (genes with an FDR q value <0.05 and log fold change >1.5 are considered significant). Abbreviations: nCRT, neoadjuvant CRT; nCT, neoadjuvant CT; TIL, tumour infiltrating lymphocyte.

## Slide 13
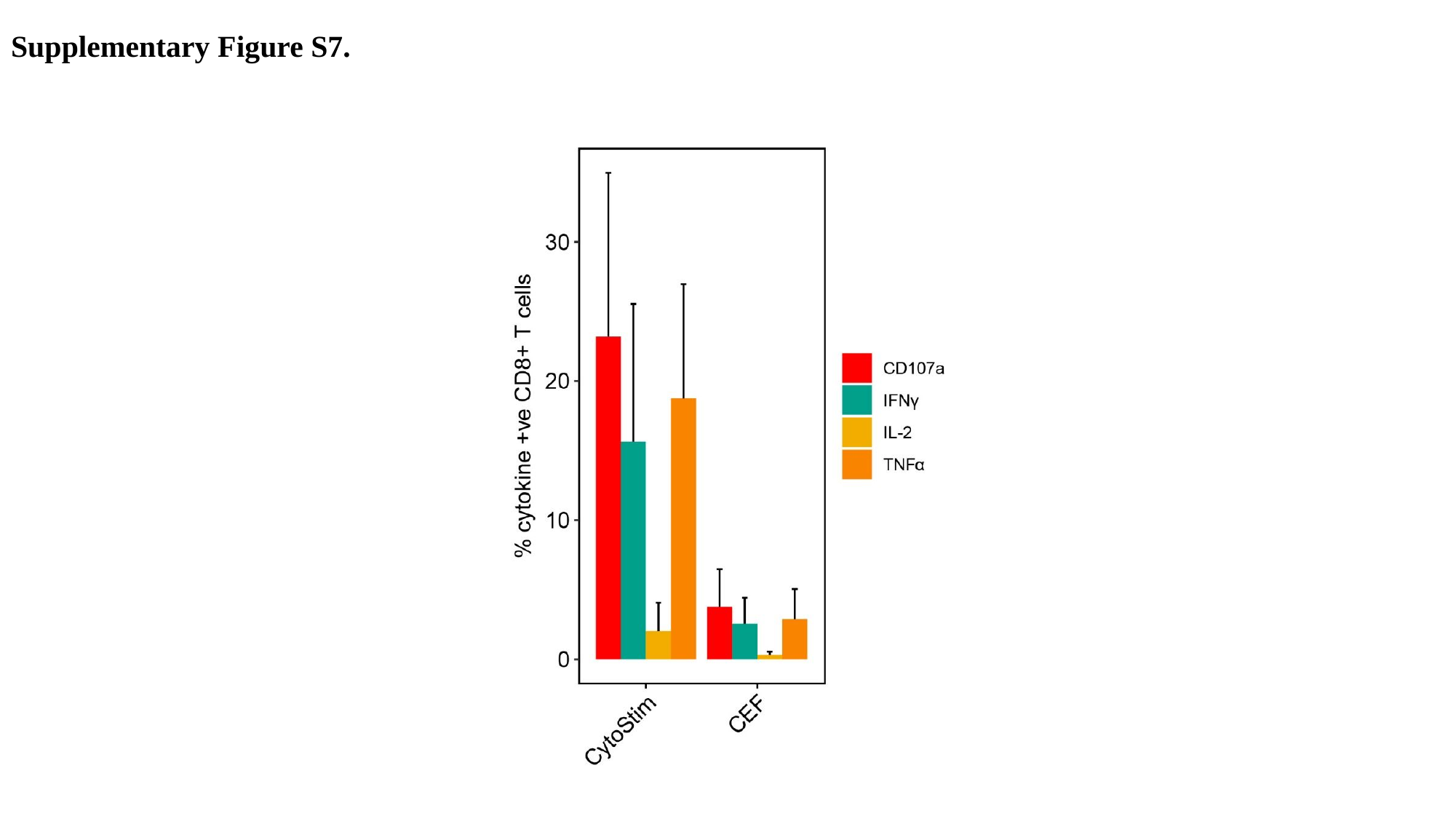

# Supplementary Figure S7.

## Slide 14
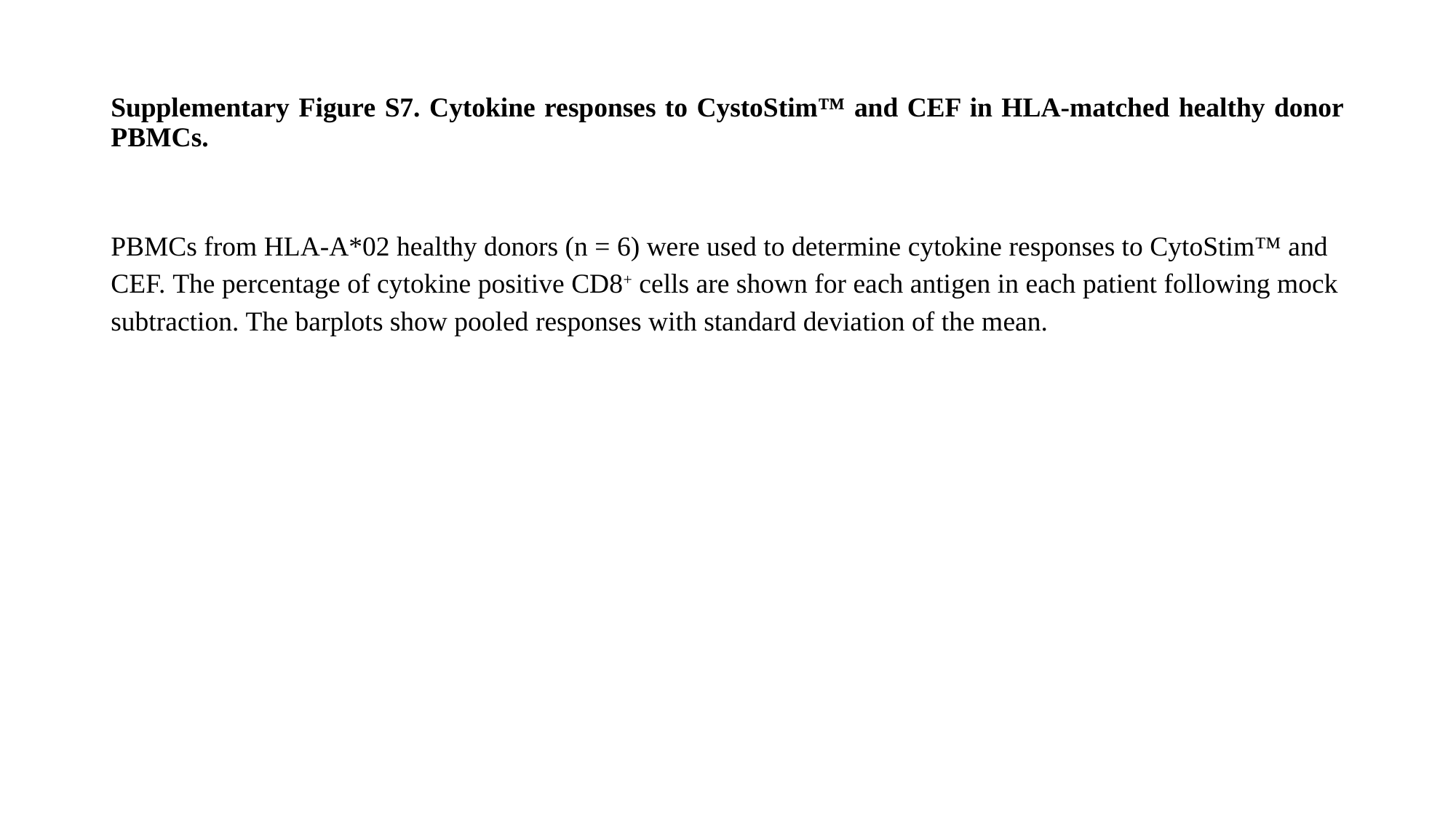

# Supplementary Figure S7. Cytokine responses to CystoStim™ and CEF in HLA-matched healthy donor PBMCs.
PBMCs from HLA-A*02 healthy donors (n = 6) were used to determine cytokine responses to CytoStim™ and CEF. The percentage of cytokine positive CD8+ cells are shown for each antigen in each patient following mock subtraction. The barplots show pooled responses with standard deviation of the mean.
