# Supplementary material for: The oesophageal adenocarcinoma tumour immune microenvironment dictates outcomes with different modalities of neoadjuvant therapy – results from the AGITG DOCTOR trial and the cancer evolution biobank
Source: Front Immunol. 2023 Oct 12;14:1220129. doi: 10.3389/fimmu.2023.1220129 (PMC10642165; doi:10.3389/fimmu.2023.1220129)
Supplement: Supplementary file 2 [file Table_1.docx]

**Supplementary Table S1**. Cohort Characteristics.

| Characteristic | Overall (*n* = 107) |
| --- | --- |
| Cohort |  |
| Cancer Evolution Biobank | 39 (36.4%) |
| AGITG DOCTOR Trial | 68 (63.6%) |
| Age at diagnosis | 61 (40 – 77) |
| Sex |  |
| Female | 14 (13.1%) |
| Male | 93 (86.9%) |
| Tumour location |  |
| Oesophagus | 59 (55.1%) |
| Gastroesophageal Junction | 48 (44.9%) |
| Clinical stage |  |
| I | 1 (0.9%) |
| II | 11(10.3%) |
| III | 77 (72.0%) |
| IV | 15 (14.0%) |
| Unknown | 3 (2.8%) |
| Neoadjuvant therapy |  |
| Chemoradiotherapy | 54 (50.5%) |
| Chemotherapy | 53 (49.5%) |
| Pathological T stage |  |
| T0 | 19 (17.8%) |
| T1 | 26 (24.3%) |
| T2 | 12 (11.2%) |
| T3 | 47 (43.9%) |
| T4 | 2 (1.9%) |
| Unknown | 1 (0.9%) |
| Pathological N stage |  |
| N0 | 63 (58.9%) |
| N1 | 19 (17.8%) |
| N2 | 13 (12.1%) |
| N3 | 11 (10.3%) |
| Unknown | 1 (0.9%) |
| Overall pathological stage |  |
| I | 46 (43%) |
| II | 17 (15.9%) |
| III | 32 (29.9%) |
| IV | 11 (10.3%) |
| NA | 1 (0.9%) |
| Primary Tumour Response |  |
| Major (<10% residual tumour) | 37 (34.6%) |
| Minor (≥10% residual tumour) | 67 (62.6%) |
| NA/Unknown | 3 (2.8%) |
| Number of resected nodes | 22 (7 – 48) |
| Residual Nodal Disease |  |
| Yes | 43 (40.2%) |
| No | 59 (55.1%) |
| NA/Unknown | 5 (4.7%) |
| Major Pathological Response |  |
| Yes | 31 (29.0%) |
| No | 74 (69.2%) |
| NA/Unknown | 2 (1.9%) |

Values represent median (range), or frequency (%).

**Supplementary Table S2**. Maximally selected rank statistics cut offs for overall survival and progression free survival.

|  | Maxstat cut off (cells/mm^2^) | |
| --- | --- | --- |
| Marker | **Overall Survival** | **Progression Free Survival** |
| Tumour CD8^+^ | 233.2 | 240 |
| Stroma CD8^+^ | 1293 | 1293 |
| Tumour CD163^+^ | 921.1 | 921.1 |
| Stroma CD163^+^ | 755.7 | 755.7 |
| Tumour FoxP3^+^ | 150 | 251 |
| Stroma FoxP3^+^ | 59.2 | 1512 |
|  | **Maxstat cut off (% total cells)** | |
| Marker | **Overall Survival** | **Progression Free Survival** |
| TC CD8^+^ >50 µm | 3.91 | 3.91 |
| TC CD8^+^ >100 µm | 3.26 | 3.4 |
| TC CD8^+^ >150 µm | 5.63 | 1.96 |
| TC CD163^+^ >50 µm | 7.31 | 1 |
| TC CD163^+^ >100 µm | 6.53 | 1.12 |
| TC CD163^+^ >150 µm | 7.41 | 1.39 |
| TC FoxP3^+^ >50 µm | 0.34 | 1.4 |
| TC FoxP3^+^ >100 µm | 2.02 | 2.02 |
| TC FoxP3^+^ >150 µm | 2.52 | 2.52 |
| TM CD8^+^ <50 µm | 1.16 | 1.92 |
| TM CD8^+^ <100 µm | 1.66 | 2.74 |
| TM CD8^+^ <150 µm | 2.63 | 1.6 |
| TM CD163^+^ <50 µm | 2.94 | 2.45 |
| TM CD163^+^ <100 µm | 2.81 | 2.81 |
| TM CD163^+^ <150 µm | 2.81 | 2.84 |
| TM FoxP3^+^ <50 µm | 2.4 | 2.78 |
| TM FoxP3^+^ <100 µm | 2.51 | 0.99 |
| TM FoxP3^+^ <150 µm | 3.12 | 2.58 |

PFS, progression free survival; OS, overall survival; TC, tumour core; TM, tumour margin.

**Supplementary Table S3.** Progression Free Survival Outcomes for the whole cohort, neoadjuvant chemotherapy and neoadjuvant chemoradiotherapy patients.

For survival estimate cut-offs, maximally selected rank statistics were used to dichotomise patients into high and low groups (33). For PD-L1 staining a cut-off of 1% was used. For cell ratios a cut-off of 1 was used to dichotomise patients into high and low groups.

|  |  | Whole cohort | | | Chemotherapy | | | Chemoradiotherapy | | |
| --- | --- | --- | --- | --- | --- | --- | --- | --- | --- | --- |
| Marker | **Group** | ***n* =** | **Median PFS** | **P value** | ***n* =** | **Median PFS** | **P value** | ***n* =** | **Median PFS** | **P value** |
| Tumour CD8^+^ density | **High** | 45 | NA | 0.023 | 28 | NA | 0.014 | 13 | NA | 0.33 |
|  | **Low** | 50 | 21 |  | 21 | 14 |  | 33 | 26 |  |
| Stroma CD8^+^ density | **High** | 18 | NA | 0.034 | 12 | NA | 0.084 | 8 | NA | 0.33 |
|  | **Low** | 71 | 26 |  | 35 | 24 |  | 34 | 30 |  |
| Tumour CD163^+^ density | **High** | 15 | 17 | 0.039 | 11 | 14 | 0.024 | 28 | 26 | 0.064 |
|  | **Low** | 80 | 51 |  | 38 | 51 |  | 18 | NA |  |
| Stroma CD163^+^ density | **High** | 35 | NA | 0.097 | 39 | 48 | 0.22 | 36 | 30 | 0.16 |
|  | **Low** | 54 | 24 |  | 8 | 19 |  | 6 | NA |  |
| Tumour FoxP3^+^ density | **High** | 30 | NA | 0.29 | 21 | 25 | 0.4 | 11 | NA | 0.38 |
|  | **Low** | 65 | 26 |  | 28 | 26 |  | 35 | 30 |  |
| Stroma FoxP3^+^ density | **High** | 10 | NA | 0.29 | 36 | 48 | 0.16 | 18 | 19 | 0.09 |
|  | **Low** | 79 | 26 |  | 11 | 14 |  | 24 | NA |  |
| Tumour PD-L1^+^ | **>1%** | 29 | NA | 0.51 | 15 | NA | 0.08 | 14 | 22 | 0.28 |
|  | **<1%** | 66 | 30 |  | 34 | 17 |  | 32 | NA |  |
| Stroma PD-L1^+^ | **>1%** | 29 | 19 | 0.34 | 16 | 14 | 0.31 | 13 | 19 | 0.73 |
|  | **<1%** | 60 | 48 |  | 31 | 48 |  | 29 | NA |  |
| Tumour CD8^+^/CD163^+^ ratio | **>1** | 36 | NA | 0.04 | 21 | NA | 0.027 | 15 | NA | 0.48 |
|  | **<1** | 55 | 20 |  | 26 | 15 |  | 29 | 26 |  |
| Stroma CD8^+^/CD163^+^ ratio | **>1** | 32 | NA | 0.12 | 20 | 48 | 0.2 | 12 | NA | 0.23 |
|  | **<1** | 51 | 21 |  | 22 | 14 |  | 29 | 21 |  |
| Tumour CD8^+^/FoxP3^+^ ratio | **>1** | 56 | 48 | 0.77 | 29 | 48 | 0.5 | 27 | 30 | 0.75 |
|  | **<1** | 36 | 26 |  | 19 | 24 |  | 17 | NA |  |
| Stroma CD8^+^/FoxP3^+^ ratio | **>1** | 47 | NA | 0.56 | 24 | 48 | 0.65 | 23 | NA | 0.78 |
|  | **<1** | 38 | 26 |  | 20 | 24 |  | 18 | NA |  |

NA, not applicable (median survival not reached); PFS, progression free survival.
